# Inferring forest fate from demographic data: from vital rates to population dynamic models

**DOI:** 10.1098/rspb.2017.2050

**Published:** 2018-03-07

**Authors:** Jessica Needham, Cory Merow, Chia-Hao Chang-Yang, Hal Caswell, Sean M. McMahon

**Affiliations:** 1 Smithsonian Institution Forest Global Earth Observatory, Smithsonian Environmental Research Center, 647 Contees Wharf Road, Edgewater, MD 21307-0028, USA; 2 Ecology and Evolutionary Biology, Yale University, 165 Prospect Street, New Haven, CT 06511-8934, USA; 3 Institute for Biodiversity and Ecosystem Dynamics (IBED), University of Amsterdam, Science Park 904, 1098 XH Amsterdam, The Netherlands

**Keywords:** forest ecology, demography, individual-based models, integral projection models, population projections, life-history strategies

## Abstract

As population-level patterns of interest in forests emerge from individual vital rates, modelling forest dynamics requires making the link between the scales at which data are collected (individual stems) and the scales at which questions are asked (e.g. populations and communities). Structured population models (e.g. integral projection models (IPMs)) are useful tools for linking vital rates to population dynamics. However, the application of such models to forest trees remains challenging owing to features of tree life cycles, such as slow growth, long lifespan and lack of data on crucial ontogenic stages. We developed a survival model that accounts for size-dependent mortality and a growth model that characterizes individual heterogeneity. We integrated vital rate models into two types of population model; an analytically tractable form of IPM and an individual-based model (IBM) that is applied with stochastic simulations. We calculated longevities, passage times to, and occupancy time in, different life cycle stages, important metrics for understanding how demographic rates translate into patterns of forest turnover and carbon residence times. Here, we illustrate the methods for three tropical forest species with varying life-forms. Population dynamics from IPMs and IBMs matched a 34 year time series of data (albeit a snapshot of the life cycle for canopy trees) and highlight differences in life-history strategies between species. Specifically, the greater variation in growth rates within the two canopy species suggests an ability to respond to available resources, which in turn manifests as faster passage times and greater occupancy times in larger size classes. The framework presented here offers a novel and accessible approach to modelling the population dynamics of forest trees.

## 1. Introduction

Forests host the majority of terrestrial biodiversity and regulate global climate, making the need to understand forest dynamics across spatial, temporal and biological scales greater than ever [1–4]. Data from large permanent plots are increasingly available (e.g. [5,6]) and can be used to parameterize demographic models that build large-scale understanding of dynamics while retaining local-scale mechanisms, such as size-dependent competition, in their structures [7,8].

However, translating measurements of individuals into insights regarding dynamics across landscapes remains a challenge. Measurement errors can be larger than growth increments between censuses [9], data are highly skewed towards small stems of common species [10], and data on critical life-history stages are scarce [11]. Further, individual differences persist through time, resulting in a minority of trees that define much of the physical structure of the forest and produce the majority of recruits [8,12,13]. There is, therefore, a need to shift focus from population averages towards a framework that accounts for temporal correlations in individual differences.

Species-specific patterns of tree mortality determine the physical structure of the forest, the light environment that drives competition in the understorey [9,14,15], successional dynamics [16–18] and carbon turnover [19]. Survival rates have been shown to vary with tree size, species and environmental conditions [20,21]. Further, carbon residence times, largely driven by patterns of mortality, remain one of the greatest sources of uncertainty in dynamic global vegetation models (DGVMs) [3]. Fitting robust models that capture species differences, as well as changes in survival through ontogeny, is therefore critical for capturing community and ecosystem dynamics.

One difficulty in modelling whole-life-history survival is capturing senescence at large sizes given that the data are dominated by small stems showing the opposite pattern of size-dependent mortality. U-shaped mortality curves have been documented in a number of mature forests [20–22] and result from different processes influencing survival probability through ontogeny. To overcome the problem of skewed size distributions, we model survival with two functions corresponding to survival in small and large individuals.

Growth data are highly asymmetric with the majority of stems growing slowly and a small subset of consistently fast growing trees. In a closed canopy forest, these asymmetries are largely driven by competition for resources, especially light [23–25]. Many models of forest dynamics, therefore, include complex representations of light environment, including species-specific crown architecture and fluctuations in light availability [26,27]. Previous studies have also used spatial coordinates to calculate competition indices that act as proxies for direct measurements of light at the individual level [11,28,29]. To capture the asymmetry in growth rates, we show how to fit multiple distributions to increment data. The multiple distributions reflect species' responsiveness to available resources (often light), i.e. the ability to grow quickly when conditions allow.

Population models of forest trees must also account for the minority of consistently fast growing individuals that contribute disproportionately to the population growth rate [12,13,30,31] and define the structure, biomass and carbon dynamics of the forest. In typical population models, e.g. integral projection models (IPMs) (cf. [32,33]), growth at each size is represented by a distribution of increments capturing the variety of mechanisms that influence growth. Population heterogeneity, however, is not correlated through time. As a result, over long time-spans average growth will trend towards the mean; a poor reflection of how trees typically grow—usually consistently slowly with rare instances of release from growth suppression resulting in sustained periods of fast growth [31]. To address this problem, we adopt the method presented by Caswell [34], adjusting the age-by-stage matrix model for a growth-by-size IPM that describes the transitions of individuals between growth distributions.

Seed and seedling dynamics have important implications for adult abundance and spatial distributions [35,36] but data are challenging to collect and integrate into population models. Recruitment, therefore, requires further treatment in future studies and is not included here. However, in the electronic supplementary material, appendix S1, we give a brief overview of various methods that can be used to complete the life cycle where data on seedlings are available.

Depending on the system of interest and the specific questions, either IPMs or individual-based models (IBMs) (e.g. [26]) may be more appropriate. IPMs offer analytical solutions making them useful for theoretical analysis and readily calculating a wide variety of population statistics [34,37]. There is a growing literature extending the IPM framework to incorporate density dependence [38], multiple state variables [39] and environmental stochasticity [40]. However, IBMs can easily be made spatially explicit and, by extension, more easily allow interactions between individuals. Furthermore, owing to the requirement for high-resolution numerical integration when constructing IPMs for long-lived, slow-growing species (electronic supplementary material, appendix S1), depending on the computational platform, analytical solutions from large IPMs can be more time consuming than simulating with an analogous IBM. We encourage ecologists to consider both options when deciding which is more suited to the question at hand.

We demonstrate how to use permanent plot data to construct both analytically tractable IPMs and stochastic simulation based IBMs. Both IPMs and IBMs take into account size-dependent survival and temporal correlations in growth rates, enabling us to capture the subset of the population that survive to adulthood. The parametrization of these models from demographic data, combined with straightforward analytic tractability, offers the potential for spatial and temporal extrapolation, for example, inferring forest dynamics across landscapes [41], or exploring evolutionary strategies through the analysis of equilibrium dynamics [42].

## 2. Methods and results

We introduce the site and data collection protocols and then describe novel vital rate functions (growth and survival) that are used to construct IPMs and IBMs. From the population models, we calculate passage times, size-dependent life expectancies and occupancy times in each growth distribution. We compare results from the IBMs, IPMs and observed census data. Electronic supplementary material, appendix S1 includes a description of sensitivity analyses to the transition probabilities between growth distributions and recommendations for IPM specifications. Further details on IPM analysis are provided in the electronic supplementary material, appendix S2. Electronic supplementary material, appendix S3 provides a fully worked example of the workflow including R scripts. All work was carried out in R [43], with RStan (http://mc-stan.org) for Bayesian estimation of vital rate parameters.

### (a) Site and data

We used data from a 50 ha forest plot located on Barro Colardo Island (BCI), Panama (9°9′ N, 79°51′ W) [44–46] (http://www.forestgeo.si.edu). The plot consists of moist, tropical forest, of which 48 ha is undisturbed old-growth forest and 2 ha is approximately 100-year-old secondary forest [47]. More detailed descriptions of the geography and vegetation of BCI are available in Croat [48], Leigh *et al.* [49] and Leigh [50]. Within the 50 ha plot all stems greater than or equal to 1 cm diameter at breast height 1.3 m above the ground (DBH) are mapped, measured and identified to species [51]. The first censuses were conducted in 1981, 1983 and 1985 and then every 5 years. In this paper, we use the census intervals from 1990 to 2015 for construction of IPMs but compare results to the full time series of data.

To illustrate the general applicability of our methods, we chose three species of varying life-history strategies; an emergent, *Prioria copaifera*, a canopy, *Calophyllum longifolium* and an understorey species, *Garcinia intermedia*. More information on these species can be found in Condit *et al.* [52] and Lim [53]. In the census intervals between 1990 and 2015 there were 2706, 3501 and 7003 stems of *P. copaifera*, *C. longifolium* and *G. intermedia*, respectively.

### (b) Survival

To capture the change in size-dependent survival through ontogeny without model fits being influenced by highly skewed size distributions, we modelled survival with one curve describing the increase in survival probability at small sizes, and another describing survival probability at large sizes. This prevents large individuals which are crucial to forest function being treated as statistical outliers.

Survival follows a Bernoulli distribution with survival probability over a census interval, *s*, given by2.1
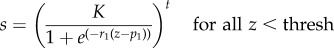
and2.2
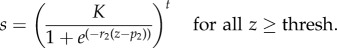


*K* is the upper asymptote of the curves, *r* and *p* are the rate of change and inflection point in the curves, respectively, with subscripts 1 and 2 denoting parameters for the small and large curves, respectively. *z* is DBH and ‘thresh’ is a species-specific size threshold at which the two curves meet. The curve is raised to the power of *t*, the time in years between censuses, so that parameters describe annual survival probability. To ensure inverted ‘u’ or flat survival forms, *r*_1_ and *r*_2_ were constrained to be ≥0 and ≤ 0, respectively, during parameter estimation. The six parameter curve (including thresh) offers sufficient flexibility to capture variation in survival rates between species, while still having interpretable parameters. For instance, *K* corresponds to survival probability over much the life cycle, *r*_1_ and *p*_1_ correspond to the degree of thinning in the understorey, and *r*_2_ and *p*_2_ correspond to senescence at large sizes.

**Figure 1.**
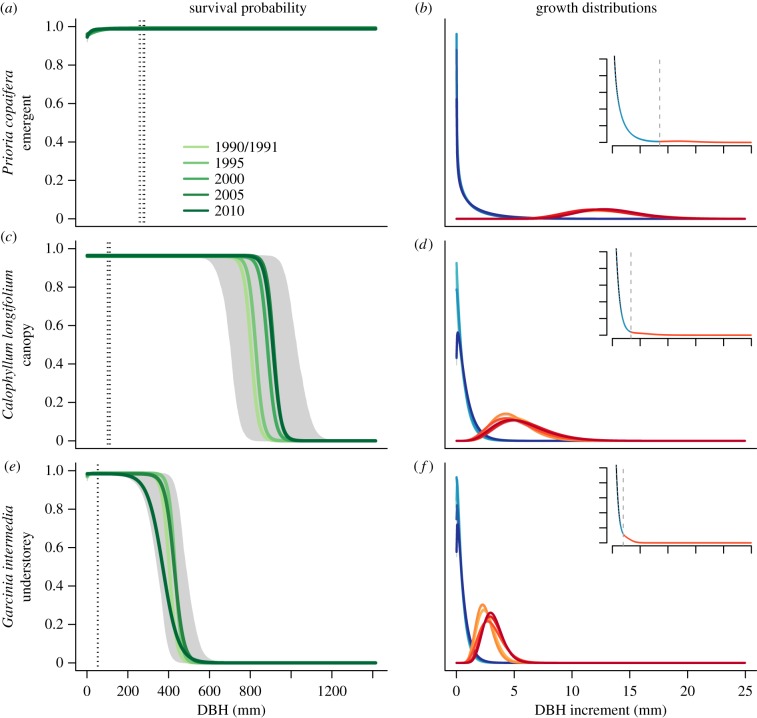
Survival and growth models for the three BCI species. Survival (*a*,*c*,*e*) is modelled with two logistic functions that meet at size thresholds shown with dotted lines (these were very similar between censuses in *G. intermedia*). Growth (*b*,*d*,*f*) is modelled with a mixture of two gamma distributions representing fast and slow absolute annual growth rates. The main plot shows the gamma distributions in each census interval, fitted to the slowest 95% of individuals (blues) and the fastest 5% of individuals (reds) (note the values on the *y*-axis are arbitrary and hence not shown). The insets show the mixed distribution, with the dotted vertical line denoting the 0.95 quantile of growth (across all censuses) and the blue and red showing the parts of the distribution we draw from when sampling slow and fast growth. Polygons show the 50th percentile of vital rate parameter estimates from the posterior distributions. Uncertainty was very low for growth parameters. The canopy species *C. longifolium* showed the greatest temporal variation in survival rates while the understorey species *G. intermedia* had the slowest growth rates.

To ensure sufficient data with which to fit each part of the curve, we fixed the size threshold as 0.25 × max(DBH). This has little influence on the curve as survival probabilities are approximately constant with size (at *K*) across much of the size range (figure 1). However, in instances where different datasets are being used to fit juvenile and adult portions of the survival curve, it might be necessary to use smoothing functions over a size interval where the two functions meet (as in [41]).

#### (i) Results: survival

*Calophyllum longifolium* had the lowest asymptotic survival rate (median over all census intervals of 0.96 compared with 0.98 and 0.99 in the other two species) (figure 1). Differences in adult survival probability of a few per cent can have significant impacts on population dynamics in long-lived species such as trees. The decreased survival of small *P. copaifera* individuals is responsible for the rapid increase in life expectancy with size early in life (electronic supplementary material, figure A.2). There was little evidence of decreased survival at small sizes in *C. longifollium* and *G. intermedia*, most probably because thinning occurs before saplings reach census size (10 mm DBH). Survival rates appeared to be temporally stable, the exception being size at senescence in *C. longifolium* and *G. intermedia* with values of *p*_2_ ranging from 804–915 and 372–431, respectively.

### (c) Growth

We modelled growth with a mixture of two gamma distributions fitted to the slowest 95% and fastest 5% of trees. Distinguishing between these common and extreme growth rates captures the way that individuals of different species perform when given access to resources, especially light. Gamma distributions offer the flexibility needed to capture the highly skewed distribution of slow growers and the more symmetric distribution of fast growers. Five per cent was chosen based on rough estimates of light availability (gap frequency, sun flecks, etc.) in closed canopy forests [54,55].

We estimated parameters for each distribution as follows:2.3
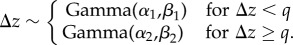


Δ*z* is absolute change in DBH, annualized by dividing change in DBH by time in years between measurements. *α* and *β* are the distribution shape and rate parameters with subscripts 1 and 2 referring to the slow and fast distributions, respectively. *q* is some percentile of observed Δ*z* (here 95%) that determines the proportion of stems that are used to fit each distribution. The two distributions were mixed with a weighting of 95% and 5% (see the electronic supplementary material, appendix S3), from which we were able to draw random samples (for simulating growth in the IBM), and estimate transitions between sizes (in construction of IPMs).

Growth is often log transformed for analyses in order to capture the multiplicative aspect of growth (as carbon gain scales as a power function [56–58]), to normalize data (which are often heavily right skewed), or to control for size differences between individuals. However, in our approach which focuses on predicting and projecting the complete life cycle, we found that transformations skew the ways in which trees are projected through long time-spans, resulting in a failure to capture realistic individual trajectories.

#### (i) Results: growth

The canopy species had faster growth rates than the understorey species *G. intermedia* (figure 1; electronic supplementary material, table A.2). The expectation of growth in each distribution, given by the medians of the slow and fast portions of the mixed gamma distribution, were 0.33 mm yr^−1^ and 2.55 mm yr^−1^, respectively, in *G. intermedia*. In comparison, these values were 0.69 and 12.80 mm yr^−1^ for the emergent species *P. copaifera*. As an understorey species, *G. intermedia* shows a conservative strategy, as long-term competition for canopy space has not been selected. On the other hand, *P. copaifera* individuals have the potential to reach the canopy and growth rates, therefore, reflect aggressive allocation to growth when resources allow.

### (d) Transitions between growth distributions

Because the two growth distributions reflect growth when individuals are suppressed or released, such as under closed canopy or in a light gap, reconstructing life-trajectories requires moving individuals between the slow and fast growth distributions. However, estimating the transition probabilities between growth distributions is difficult owing to long census intervals, and context-dependent canopy dynamics.

Spatial information could be used to estimate changes in individuals' competitive neighbourhoods, although this requires finding relevant estimates of neighbourhoods that correlate with growth rates. With annual growth data, a hidden Markov model (HMM) would allow simultaneous estimation of growth parameters and transition probabilities between growth distributions. The growth distribution of each individual would be a latent state which could be estimated based on observed size at each time step. However, with 5 year census intervals, HMMs are non-identifiable, as many combinations of annual growth can produce the same 5 year growth rate (see the electronic supplementary material, appendix S1).

We explored transitions in two ways. First, we built models (IPMs and IBMs) without transitions, to demonstrate for each species the slowest and fastest lifetime trajectories. We then explored an IBM example with size-dependent transitions between growth distributions representing the growth response to light attenuation through the canopy. With increasing DBH, trees are assumed to be taller and have increased access to light (see fig. 5, [55]), resulting in an increased probability of growing fast. The probabilities of moving from slow to fast, and of remaining fast, increased linearly from 0.1 at the smallest size to 0.99 at the largest size. Probabilities of remaining slow or moving from fast to slow were the compliment of this. Second, we conducted a sensitivity analysis of IPM passage times and life expectancies to transition probabilities (see the electronic supplementary material, appendix S1).

### (e) Vec-permutation integral projection models

IPMs in which individuals are classified by size and growth distribution require a vec-permutation approach to link these two states [34,59]. The IPMs we develop combine matrices describing growth and survival within a growth distribution, with matrices describing transition probabilities between growth distributions, analogous to the age by stage matrix models in Caswell [34], but with growth distribution replacing age. As the theory for vec-permutation population models has already been presented in detail [34], we describe construction of such models in the electronic supplementary material, appendix S1 and a fully worked example with R code in the electronic supplementary material, appendix S3.

We constructed IPMs and calculated passage times to 200 mm DBH, i.e. the number of years an individual of each size and starting growth distribution would be expected to take to grow to 200 mm DBH, conditional on survival. We also calculated longevity, i.e. the expected number of years until death at each size, and the expected occupancy time in each growth distribution for stems above and below 200 mm DBH. See the electronic supplementary material, appendix S2 for more details on how these metrics are calculated, and electronic supplementary material, appendix S1 for a sensitivity analysis of these outputs to the number of size bins used to construct the IPMs.

#### (i) Results: integral projection models

Passage times to 200 mm DBH were shorter for the two canopy species as a result of faster growth rates. Life expectancy early in life was lowest in *C. longifollium* as a result of the lower long-term survival probability (*K*). Despite having the fastest growth rates, *P. copaifera* had the longest occupancy times at sizes above 200 mm DBH, owing to high asymptotic survival and late senescence. Results are described in more detail in the electronic supplementary material, appendix S1 (figures A.2 and A.3).

### (f) Individual-based models

We ran an IBM to compare individual trajectories to census data. Each year of the IBM simulation, individuals survive according to a draw from a binomial, with size-dependent survival probabilities given by the species-specific survival function, equations (2.1) and (2.2). Survivors grow according to a random draw from the mixed gamma distribution equation (2.3). Growth increments are sampled from above or below the increment threshold of the mixed distribution (defined as the 95% percentile of growth increments from all censuses combined), according to whether an individual is in fast or slow growth. Individuals can then potentially move between growth distributions. We initialized the IBM with the size distribution of each species in 1981 and projected forward 500 years. We compared estimates of passage time to 50 mm DBH from our IPMs and IBMs with the mean time taken to reach 50 mm DBH for stems less than or equal to 20 mm DBH in 1981–1983 in the census data.

#### (i) Results: individual-based models

**Figure 2.**
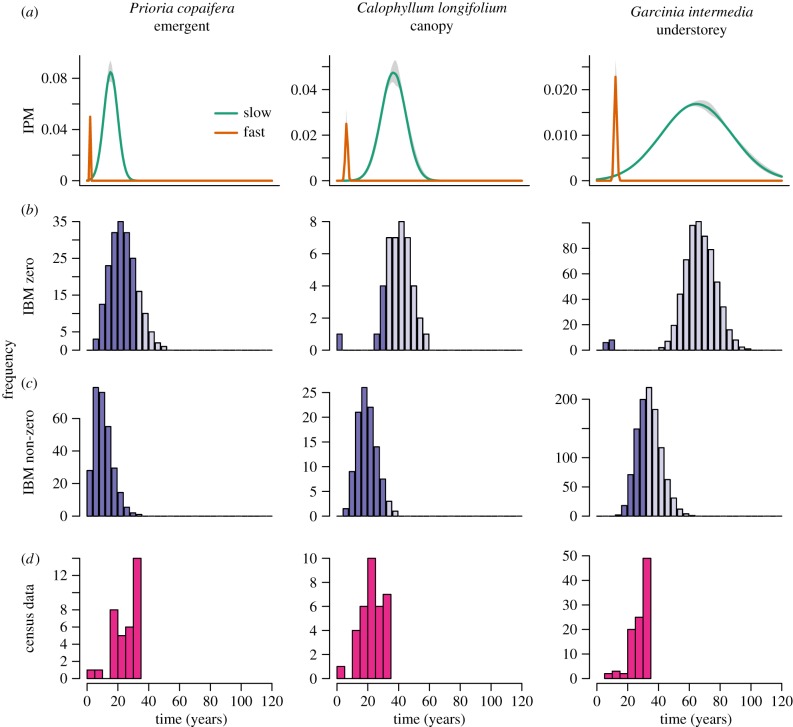
Passage times of each species from 10 mm to 50 mm DBH, estimated from IPMs (*a*), IBMs with zero transitions between growth distributions (*b*), IBMs with non-zero size-dependent transitions between growth distributions (*c*), and the census data (*d*). Lighter and darker histograms show the distribution of passage times from the full 500 years of IBM simulations and when results were truncated to 34 years to match the census data time series. Passage times were estimated from the census data by tracking how long it took individuals between 10 and 20 mm DBH in 1981 to reach 50 mm by 2015. Estimates from the census data show a truncated distribution, as many individuals will take longer than 34 years to reach 50 mm. Results best matched the census data when transitions were zero for *P. copaifers* but when size-dependent in the other two species.

The distribution of passage times to 50 mm DBH from the IBMs and IPMs were, for the most part, qualitatively similar to census data for each species (figure 2; electronic supplementary material, A.8), considering the data are temporally truncated. When transition probabilities between growth distributions were zero, the distribution of passage times from IPMs and IBMs most closely matched observations in the emergent species *P. copaifera*. Observed passage times to 50 mm DBH had a minimum of 4 and a median of 29 years, while the minimum and median passage times from the IBMs were 4 and 26 years, respectively, and passage times of fast and slow growers were 3 and 16 years in the IPMs. *Calophyllum longifolium* and *G. intermedia* dynamics more closely matched census data when transition probabilities between growth distributions changed in a size-dependent manner. For example, the minimum and median passage times from the census data were 4 and 24 years for *C. longifolium* and 5 and 23 years from the IBM. In *G. intermedia*, minimum and median passage times in the census data were 10 and 30 years, and 13 and 37 years in the IBM.

It is worth noting that in the field data very few individuals grew to 50 mm DBH within the 34 year time frame, and those that did represent the very fastest growing individuals in the population. With longer time series of data we expect the median passage time to a given size to increase, i.e. the right-hand side of the distribution to be revealed.

## 3. Discussion

Capturing demographic rates, and their impact on population dynamics, is a critical step in advancing our understanding of how forests will respond to global change. Here, we show how individual level measurements can be scaled up to provide population-level inference.

We find that models accounting for persistent variation in individual vital rates are able to reproduce observed dynamics of three tropical tree species with differing life histories. However, validating predictions of passage times and life expectancies is challenging given that few, if any, datasets extend over the full life cycle of canopy trees. Here, we were able to compare results from our projections to a 34 year time series of data, but although results matched observed trajectories of individuals, this was still only a small proportion of the life cycle for all three species. Brienen & Zuidema [31] used tree ring data to study lifetime growth patterns of tropical trees and found considerable variation within and between species. However, in the tropics many species do not produce tree rings, and when they do, samples are biased towards trees that survived to reach adult stature. Passage times calculated from IPMs or IBMs on the other hand provide information on the full distribution of pathways through the life cycle (figure 2).

### (a) Harnessing demographic data for use in forward projections

Forest plot data are available from all the major forest biomes globally, e.g. [5,6,60,61], allowing statistical descriptions of size-dependent vital rates (e.g. [62]). Although the methods presented here can be implemented using only census data, the modular nature of the workflow makes it easy to extend any or all of the vital rate models, e.g. by including environmental covariates [41,63] or interactions between vital rates. The increased probability of mortality associated with slow growth has been noted for many years [64] and could be incorporated here, either through parametrization of separate survival functions for each growth distribution, or by making survival probability a function of both size and growth rate.

Although ideally we would fit mechanistic models based on detailed understanding of the physiological processes that drive individual growth, survival and reproduction, the data necessary to parameterize such models are generally not available across broad scales. Further, recent studies have found that gap models with simple representations of vital rates are able to reproduce the dynamics predicted by more complex models [65]. While additional parameters improve the fit of a model to a given dataset, they narrow the applicability of the model [66] and, in this context, complicate efforts at forward projection.

### (b) Temporal correlations in vital rates

There are many examples of population models that incorporate temporal correlations in vital rates (e.g. [67,68]), but examples for tree species often rely on data which are not commonly available, for example, measurement of individual light environments [22] or individual age [12]. An alternative approach is to model ‘individual effects’, e.g. through inclusion of individual intercepts in regressions of vital rates. Individual ‘quality’ can either be fixed [39], or modelled with linear autoregression so that quality at each time step depends linearly on quality at the time step before [37, p. 162–167]. In our framework, individuals move between growth distributions (the equivalent of quality) in a size-dependent manner, that is independent of previous growth. The Ellner *et al.* [37] approach suits cases where quality is a continually varying state. However, we find that two growth distributions work well for trees, where access to resources, especially light, result in large asymmetries in growth rates within the population. Other forest dynamics models also deal with the binary nature of light by discretely dividing populations. For instance in the perfect plasticity approximation [24,69] individuals are either in or out of the canopy, and two sets of vital rates govern dynamics of understorey and canopy trees.

### (c) Applications and future directions

Biodiversity needs to be better represented in global climate models. DGVMs diverge widely in their predictions of the feedback between vegetation and changing climates (e.g. [3,70,71]), partly because they move from physiology to global dynamics with very little consideration of the scales in between (e.g. species or communities) (e.g. [3,72]). Yet, carbon uptake and residence times are driven by species-specific patterns of mortality and growth at the landscape level (e.g. [73,74]). This calls for models that take into account how individual level processes scale to demographic rates and how within- and between- species differences influence community composition and carbon turnover [1–4,21]. Advances in terrestrial and airborne lidar systems have made it possible to track the fate of individual trees [75]. Integrating remote sensing technologies with forest inventory data will be one way to track demography across landscapes.

### (d) Conclusion

Species differences in demographic rates are critical to determining the outcome of local competitive interactions. Over large extents these competitive interactions determine species range shifts and shape community composition. Yet, moving from individual level data, through descriptions of vital rates and population models to an understanding of forest dynamics across large geographical and temporal scales remains an important challenge. By proposing flexible vital rate models that capture the critical processes shaping populations, we make best use of increasingly available forest inventory data to translate knowledge of individuals into an understanding of dynamics at the population level. This demographic approach will be essential for reducing uncertainty in predictions of how forests will respond to global change.

## Supplementary Material

Appendix 1: Guidance for population model construction

## Supplementary Material

Appendix 2: Theory for Vec-Permutation IPM analysis

## Supplementary Material

Appendix 3: R scripts for the complete workflow

## Data accessibility

Data are available upon request from the CTFS-ForestGEO website at http://www.forestgeo.si.edu. [46].
