# Supplementary material for: Inferring forest fate from demographic data: from vital rates to population dynamic models
Source: Proc Biol Sci. 2018 Mar 7;285(1874):20172050. doi: 10.1098/rspb.2017.2050 (PMC5879618; doi:10.1098/rspb.2017.2050)
Supplement: Appendix 1: Guidance for population model construction [file rspb20172050supp1.pdf]

Inferring forest fate from demographic data: from vital rates to population dynamic models: Appendix 1

Jessica Needham[1], Cory Merow[2], Chia-Hao Chang-Yang[1], Hal Caswell[3], and Sean M. McMahon[1]

[1]Smithsonian Institution Forest Global Earth Observatory, Smithsonian Environmental Research Center, 647 Contees Wharf Road, Edgewater, MD 21307-0028

[2]Ecology and Evolutionary Biology, Yale University, 165 Prospect St, New Haven, CT 06511-8934,

[3]Institute for Biodiversity and Ecosystem Dynamics (IBED), University of Amsterdam, Science Park 904, 1098 XH Amsterdam, The Netherlands

Contents

1 Growth correction **2**

2 Vec-permutation IPM construction **2**

3 Passage times, longevities and occupancy time results **4**

4 Sensitivity analyses **4**

5 Inclusion of recruitment **6**

6 Eviction of individuals from IPMs **7**

7 Recommendations for IPM specifications **7**

8 Latent Resource State Model **8**

9 Figures **9**

10 Tables **17**

1 Growth correction

A few modelling decisions were helpful for fitting sensible growth distributions. Prior to fitting the growth model we made growth increments positive using an adapted version of the method presented by Rüger *et al.* (2011). With five year census intervals, negative increments are most likely to result from measurement errors as opposed to physiological mechanisms. We therefore proposed new sizes for each individual based on estimates of the frequency and magnitude of measurement error (Rüger *et al.*, 2011), until growth increments were positive for all stems. Details on growth correction are provided in Appendix 3. To avoid introducing bias by only adjusting negative growth, we applied the same correction to the positive increments, such that growth increments were adjusted for all stems.

2 Vec-permutation IPM construction

To be consistent with Caswell (2012), we use matrix notation to describe construction and analysis of IPMs. However, it is worth noting that the matrices represent IPM kernels in which size is continuous (Easterling *et al.*, 2000). In IPMs, size is only discretised in order to numerically solve the integration of transition probabilities between sizes.

We begin by making matrices which describe the probability of growth from every size to every other size, combined with the survival probability at each size. There is a growth and survival matrix, $\mathbf{P}$, for every growth distribution. If we have discretised size into S size bins, and there are G growth distributions (here 2) then we have $\mathbf{P}_1$, $\mathbf{P}_2 \dots \mathbf{P}_G$ with each $\mathbf{P}$ matrix of dimensions S x S (note that our G growth distributions replace the i age classes used in Caswell (2012)). These $\mathbf{P}_{1\dots G}$ matrices are arranged in a block diagonal matrix $\mathbb{P}$.

$$\mathbb{P} = \begin{pmatrix} \mathbf{P}_1 & 0 & \dots & 0 \\ 0 & \mathbf{P}_2 & \dots & 0 \\ \vdots & \vdots & \ddots & \vdots \\ 0 & 0 & \dots & \mathbf{P}_G \end{pmatrix}$$

To describe the transition probabilities of individuals *between* growth distributions we construct a G x G matrix, C for every size bin S and arrange them in a block diagonal matrix $\mathbb{M}$, of dimensions GS x GS (in Caswell (2012) this is referred to as $\mathbb{D}_U$).

To enable comparison of extremes of trajectories through the life-cycle we fixed transition probabilities between growth distributions at 0, such that individuals were consistently either slow or fast over the entire lifetime. However, transitions between growth distributions could follow a range of size-dependent or independent rule sets.

We next make $\mathbf{T}$, the vec-permutation matrix (called K in (Caswell, 2012)). $\mathbf{T}$ is of dimension GS x GS and consists of 1s and 0s. It rearranges a column vector holding the size distribution of the population, $\tilde{\mathbf{n}}$, from (in the case of $G = 2$, shown here transposed)

$$\tilde{\mathbf{n}}_t = \left(\overbrace{n_{1,1},\ n_{2,1}, \ldots n_{S,1}}^{G_1} \middle| \overbrace{n_{1,2},\ n_{2,2} \ldots n_{S,2}}^{G_2} \right)^{\mathsf{T}}$$

where individuals are arranged as sizes within growth distributions, to

$$\mathbf{T}\tilde{\mathbf{n}}_t = \left(\overbrace{n_{1,1},\ n_{1,2}}^{S_1} \middle| \overbrace{n_{2,1},\ n_{2,2}}^{S_2} \middle| \ldots \middle| \overbrace{n_{S,1},\ n_{S,2}}^{S_S} \right)^{\mathsf{T}}$$

where individuals are arranged by growth distributions within size classes.

The final growth and transition matrix $\tilde{\mathbf{P}}$ is given by

$$\tilde{\mathbf{P}} = \mathbf{T}^{\mathsf{T}}\mathbb{M}\mathbf{T}\mathbb{P}. \tag{1}$$

Reading eqn. (1) from right to left, $\mathbb{P}$ first moves individuals between size classes within a growth distribution. $\mathbf{T}$ rearranges the population vector so that $\mathbb{M}$ can move individuals between growth distributions. Finally $\mathbf{T}^{\mathsf{T}}$ rearranges the population vector back to its original orientation.

$\tilde{\mathbf{P}}$ is sufficient to enable analysis of cohort dynamics, such as calculating passage time to a certain size or expected longevity of each size class. Where data on per capita reproduction is available, it is also possible to construct the fecundity matrix $\tilde{\mathbf{F}}$ which allows analysis of full population dynamics. Modelling recruit production was beyond the scope of the paper, but for completeness we include the method for constructing $\tilde{\mathbf{F}}$ below.

$\tilde{\mathbf{F}}$ is constructed much as $\tilde{\mathbf{P}}$, with a series of $\mathbf{F}$ matrices, $\mathbf{F}_1$, $\mathbf{F}_2 \ldots \mathbf{F}_G$, describing production of offspring of each size by adults of each size, in each growth distribution G. These are arranged in a block diagonal matrix $\mathbb{F}$. The assignment of offspring into each growth distribution, by adults in each growth distribution, is given by $\mathbb{D}$.

The $\tilde{\mathbf{F}}$ matrix is given by

$$\tilde{\mathbf{F}} = \mathbf{T}^{\mathsf{T}}\mathbb{D}\mathbf{T}\mathbb{F}. \tag{2}$$

Finally $\tilde{\mathbf{K}}$, the full IPM matrix describing growth, survival and fecundity, along with movement of individuals between growth distribution is given by

$$\tilde{\mathbf{K}} = \tilde{\mathbf{P}} + \tilde{\mathbf{F}}. \tag{3}$$

3 Passage times, longevities and occupancy time results

Passage times to 200 mm DBH showed similar qualitative patters in all three species, (Fig. A.2). However, the faster growth rates of the canopy species resulted in mean passage times of 14 and 34 years for 10 mm DBH stems starting in the fast growth distribution in *P. copaifera* and *C. longifolium* respectively, compared with 61 years for *G. intermedia* individuals.

Life expectancies were highest in *P. copaifera* at all sizes (Fig. A.2). The higher longterm mortality of *C. longifolium* resulted in life expectancies being higher in the understory shrub *G. intermedia* than in *C. longifolium* at 10 mm DBH (50 versus 26 years, starting fast), despite the potential of *C. longifolium* to reach much larger sizes.

Expected occupancy in each size range and growth distribution combination are shown in Fig. A.3. With transition probabilities between growth distributions set to 0, the expected occupancy in each growth distribution equals the life expectancy of individuals in that growth distribution. However, with movement of individuals between growth distributions, occupancy times can provide information on time spent in combinations of size ranges and growth distributions. This is useful, for example, in estimating time spent in gaps versus shade for understory individuals, or how growth rates influence time spent at reproductive sizes.

Because transition probabilities within the IPM depend only on current state and not how an individual got there, survival probability is size but not age dependent. Starting growth distribution therefore has little influence on occupancy times at large sizes. In reality, it is possible that individuals that take longer to reach the canopy might have lower survival once there, due to factors such as accumulated pathogen load and herbivore damage (Ireland *et al.*, 2014).

4 Sensitivity analyses

Transitions between growth distributions We tested the sensitivity of IPM outputs to size-dependent transition probabilities between growth distributions by constructing IPMs with different combinations of transition probabilities from slow to fast and fast to slow. Size-dependent transition probabilities are described by a linear function. In this sensitivity analysis, we fixed the probability of moving from slow to fast at 0.99 at the largest size and altered the transition probability at the smallest size from 0 to 0.9, thus creating a range of gradients for the size dependency of transitions (shown in figs A4-A6 top panels). For simplicity we made the probability of remaining in the fast distribution equal to the probability of moving from slow to fast. The probabilities of remaining slow, or moving from fast to slow are the compliment of these. From each combination of slow to fast and fast to fast parameters, we constructed IPMs and calculated passage times to 200 mm DBH and size-dependent life expectancy,

conditional on growth distribution. Sensitivities to transition probabilities were tested with all three species using IPMs with S = 1000.

Results In all three species, as expected, passage times were shortest when the probabilities of moving into the fast growth distribution (slow to fast) and remaining there (fast to fast) were highest at small sizes. Generally, passage times were more sensitive to the slow to fast transitions than to fast to slow transitions (with the exception of *P. copaifera*). Life expectancies showed the same qualitative pattern but tended to be less sensitive to transition probabilities than passage times (figs A.4, A.5 and A.6).

For a given probability of moving from slow to fast, changing the fast to fast transitions led to changes in passage times from 10 to 200 mm DBH of up to 35 years (24 to 59 years), in *P. copaifera*. As the probability of transitioning from slow to fast at small sizes increased, the effect of changing fast to fast transitions decreased.

Life expectancies for 10 mm DBH *P. copaifera* individuals ranged from 72 to 82 years for individuals starting in the fast growth distribution and 67 to 79 years for individuals starting in the slow growth distribution. Life expectancies in the other two species were less sensitive to transition probabilities, possibly due to shorter expected life spans over which these differences can play out, and smaller differences in growth rates between the two distributions. However, passage times were still sensitive to transitions in these species, ranging from 36 to 110 years for 10 mm DBH *C. longifolium* stems starting in the fast growth distribution to reach 200 mm DBH, and 69 to 154 years for *G. intermedia* stems (Figs A.5 and A.6).

Sensitivity to matrix size

The dimensions of IPMs determine the accuracy with which we can estimate population statistics. In IPMs, the state variable (size) against which vital rates are regressed is treated as continuous, but numerical integration involves discretisation of the size variable into a large number of discrete bins. Because trees typically grow a very small amount each year, relative to their potential maximum size, a large number of size bins are needed to capture individual heterogeneity in growth and prevent the unrealistically fast movement of some individuals through the size classes (Zuidema *et al.*, 2010). However, the time taken to analyse a matrix increases with the size of the matrix. In multi-state IPMs, the dimensions of the matrix are given by the product of the number of classes in each state, for example the number of size classes, multiplied by the number of growth distributions. Increasing the number of state variables, and the number of classes in each, can quickly make calculating metrics such as the population growth rate–the dominant eigenvalue–prohibitively slow. We therefore test the sensitivity of population level outputs from our size-growth IPMs to the number of size classes, since a reduced number of size classes increases the efficiency with which further state variables can be added to the IPMs.

We tested the sensitivity of population statistics for all three BCI species to the number of size bins

(S), by constructing IPMs from S = 200, to S = 2000 in increments of 200 and as well as IPMs with S = 3000 and S = 4000. Corresponding size class widths ranged from 8.46 mm to 0.42 mm, 3.75 mm to 0.19 mm and 1.62 mm to 0.08 mm in *P. copaifera*, *C. longifolium* and *G. intermedia* respectively. From each IPM we calculated longevities, and passage times to 200 mm DBH, conditional on starting growth distribution.

Results Passage times from 10 to 200 mm DBH increased for slow growing individuals in all three species as the number of size bins increased, but remained fairly constant for fast growing individuals. Life expectancies were more insensitive to IPM dimensions, decreasing for fast growers and increasing for slow growers with increasing size bins in *P. copaifera* but remaining relatively unchanged in *C. longifolium* and *G. intermedia*.

Estimates of passage time for a 10 mm *P. copaifera* stem, starting in the slow growth class, to reach 200 mm varied from 22 to 118 years when size bins ranged from 200 to 4000 (Fig. A.7). In contrast passage times for fast growing individual only ranged from 11 to 15 years. Life expectancies ranged from 88 to 71 years for a 10 mm stem in slow growth, as the size bins increased from 200 to 4000.

Calophyllum longifolium life expectancy was insensitive to matrix size, not varying from 25 years as size bins ranged from 200 to 4000. Passage times were more sensitive to matrix size, ranging from 50 to 230 years for a 10 mm DBH stem to reach 200 mm DBH starting in the slow growth (Fig. A.7). *Garcinia intermedia* passage times at 10 mm to 200 mm DBH ranged from 117 to 393 years in slow growth, (Fig. A.7). The range in passage times for fast growing individuals of these two species was just 13 and 15 years respectively.

5 Inclusion of recruitment

Several features of tree reproductive strategies make modelling reproduction and recruitment a challenge. The relationship between size and seed production is not always clear and as a result inverse models of seed production have been used to infer species specific reproductive size thresholds and size-fecundity relationships (Uriarte *et al.*, 2005; Muller-Landau *et al.*, 2008). Further, masting is a common among many species, but the drivers are still being elucidated (Wright & Calderón, 2006; Sun *et al.*, 2007; Pau *et al.*, 2013).

Vital rate models of seedling demography need to account for seedling specific behaviours such as shrinkage and different measurement units (typically height). We can combine the kernels of seed production, seedlings and trees into a full life cycle IPM by adjusting the vec-permutation approach (Caswell, 2012) with matrices of transition probabilities among different stages. Transitions between seedlings and trees (or saplings) might be estimated by seedling IPM simulations (Chang-Yang et al. unpublished data)

or obtained from field data.

6 Eviction of individuals from IPMs

Eviction of individuals from IPMs occurs when the range of sizes dictated by the IPM is less than the maximum that can be obtained through the interplay between the growth and survival models. This results in abrupt death of anything still in the final size bin, as by growing out of the IPM, they disappear from the population. This sudden mortality limit can lead to underestimates of population growth rates (Williams *et al.*, 2012), and failure to capture senescence of large individuals. To ensure that the rate of senescence results in individuals dying before reaching the maximum size, either the modelled size range can be extended, priors can be used when fitting the survival curve, or the survival parameters determining senescence can be sampled in an inverse model. In this inverse modelling approach, the approximate likelihood function compares simulated and observed size distributions and rejects parameters that allow individuals to survive beyond the sizes observed in the data set.

In our example, results from an inverse model would likely be similar to extrapolation from the forward model through extension of the size range, as the survival curve fit to the observed sizes has a slight dip in survival probability, which, when extrapolated, leads to senescence in sizes beyond the observed size range. When data are more limited and few mortality events are observed in large size classes, inverse modelling might be necessary to avoid high rates of survival leading to individuals surviving beyond the observed size range.

7 Recommendations for IPM specifications

Decisions regarding IPM dimensions and the number of growth distributions to use depend on the study species and the available data. Species with larger maximum sizes will generally need larger IPMs in order to have sufficient resolution to capture variation in growth rates.

There is a trade-off between precision and computation time with regards to increasing IPM size. Based on our sensitivity analysis to matrix size, we suggest approximately 4000 size bins are necessary to get good estimates of life expectancies and passage times. While life expectancies and passage time in the fast growth distribution stabilised around 800 size bins in all three species, passage times in the slow growth distribution were sensitive to matrix dimensions and failed to stabilise by 4000 size bins, although they showed signs of levelling off. It is worth noting that when transition probabilities between growth distributions are non zero, differences in passage times between fast and slow growers decrease, and sensitivities of both groups to matrix dimensions also decrease.

Our sensitivity analysis was carried out on a linux red hat x86_64 using R (R Core Team, 2017).

Alternative computers, software or R packages will obviously change the importance of the trade-off between precision and speed.

Regarding the number of growth distributions, we generally recommend sticking to two distributions. Transition probabilities between growth distributions are difficult to estimate but have a substantial impact on results. Since the number of transitions to estimate is the square of the number of growth distributions, unnecessary distributions should be avoided. Two distributions make sense biologically, and since results were generally insensitive to whether a two or three distribution model was used (results not shown) there seems to be little reason to increase the number of parameters to estimate and the computational cost of analysing the IPM.

8 Latent Resource State Model

We know that access to resources is a primary determinant of growth rates, yet the resource environment of individuals is difficult to directly measure. In our framework we assume that individuals are in one of two resources states each year (slow or fast) and growth increments are drawn from a distribution describing growth for each of those states. The challenge is to estimate how individuals transition between these resource states (which are unobserved) based only on observed sizes from census data.

In statistics, Hidden Markov Models are used to infer latent states from emitted signals. In this context the latent state is resource availability (slow or fast growth distribution) and the emitted signal is size, measured every five years. This is shown in Fig. A.1. However, a number of complexities make this a non-identifiable problem. Firstly, the aim is to estimate annual transitions, but the census data on individual sizes is collected approximately every five years. Secondly, feedbacks exist between size, growth and resource availability. Access to resources leads to faster growth rates and larger sizes, and large individuals generally have better access to resources (better developed rooting systems and larger canopies).

Due to these complications, we decided to bracket the problem and offer an example solution. First, we fixed transitions at zero, to demonstrate the shortest and longest pathways through the life-cycle for each species. We then explored how a range of size-dependent transitions influence population dynamics with a sensitivity analysis.

9 Figures

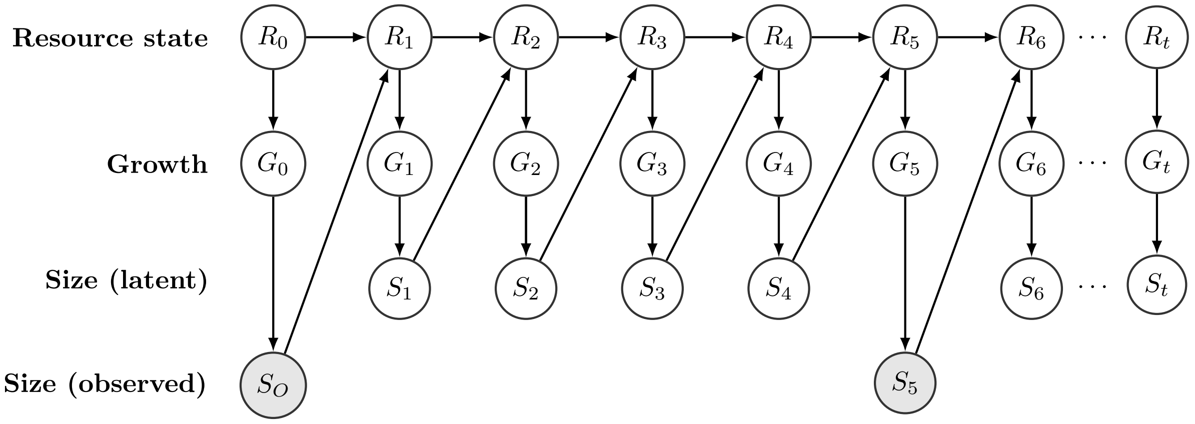

Figure A.1: HMM of tree growth response to resource availability (light). The resource state R $(0, 1)$ indicates access to light, which will determine a draw from either slow or fast growth distributions. Larger trees are more likely to be in light. Growth changes size, which affects the probability of being in light. This latent model repeats annually. The only data we have are the observed sizes of the trees every five or so years.

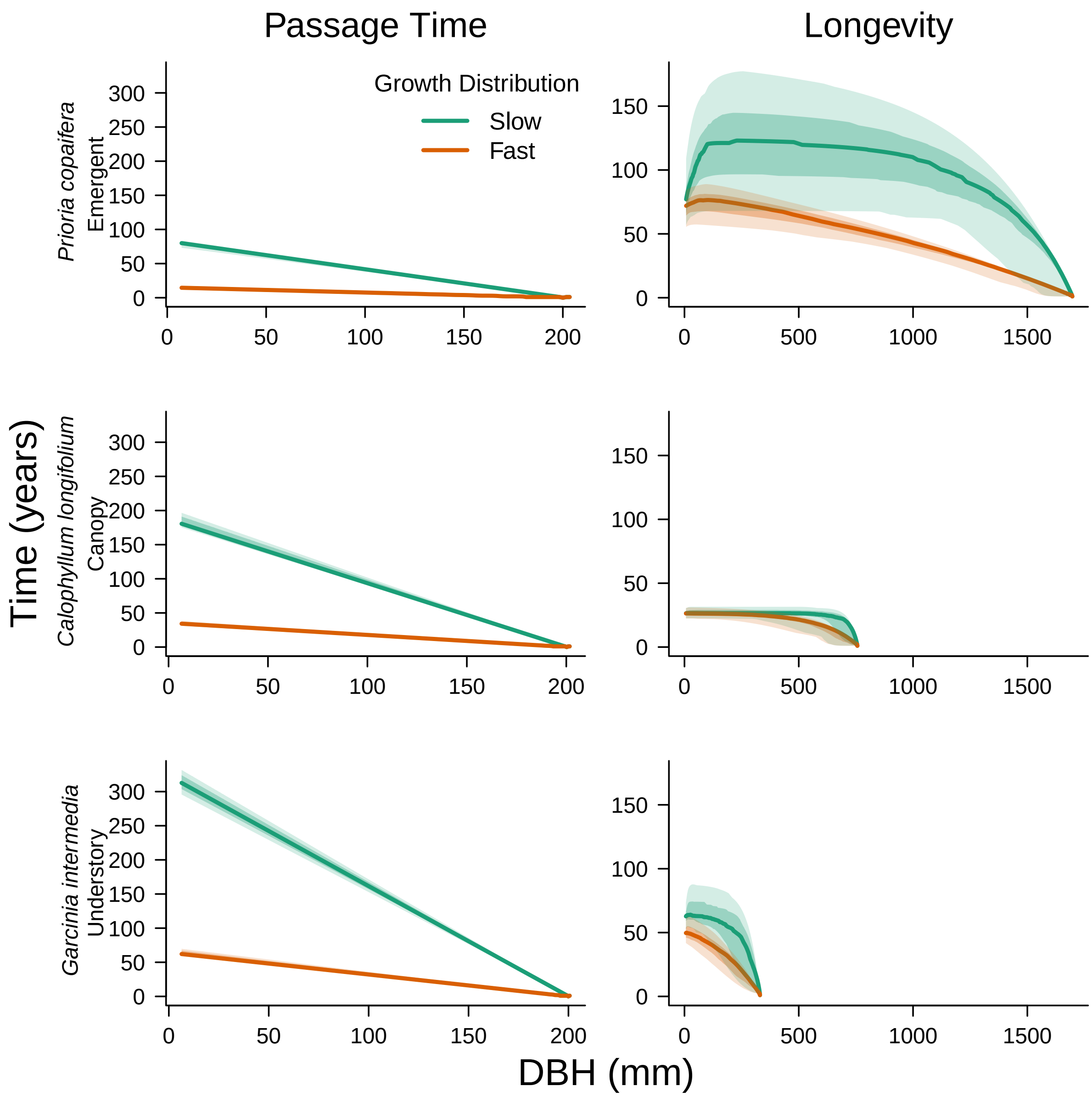

Figure A.2: Passage times to 200 mm DBH (left) and life expectancies (right) for the BCI species, conditional on growth distribution. Lighter polygons show the 50th and 95th percentile of uncertainty, obtained by propagating uncertainty from the posterior distributions of vital rate parameters through to the IPM, i.e. parameter uncertainty in estimates of mean passage times. Passage times were fastest for *P. copaifera* as a result of the much higher growth rates in this species. Life expectancy at the smallest size was lowest in *C. longifolium* as a result of lower overall survival of this species.

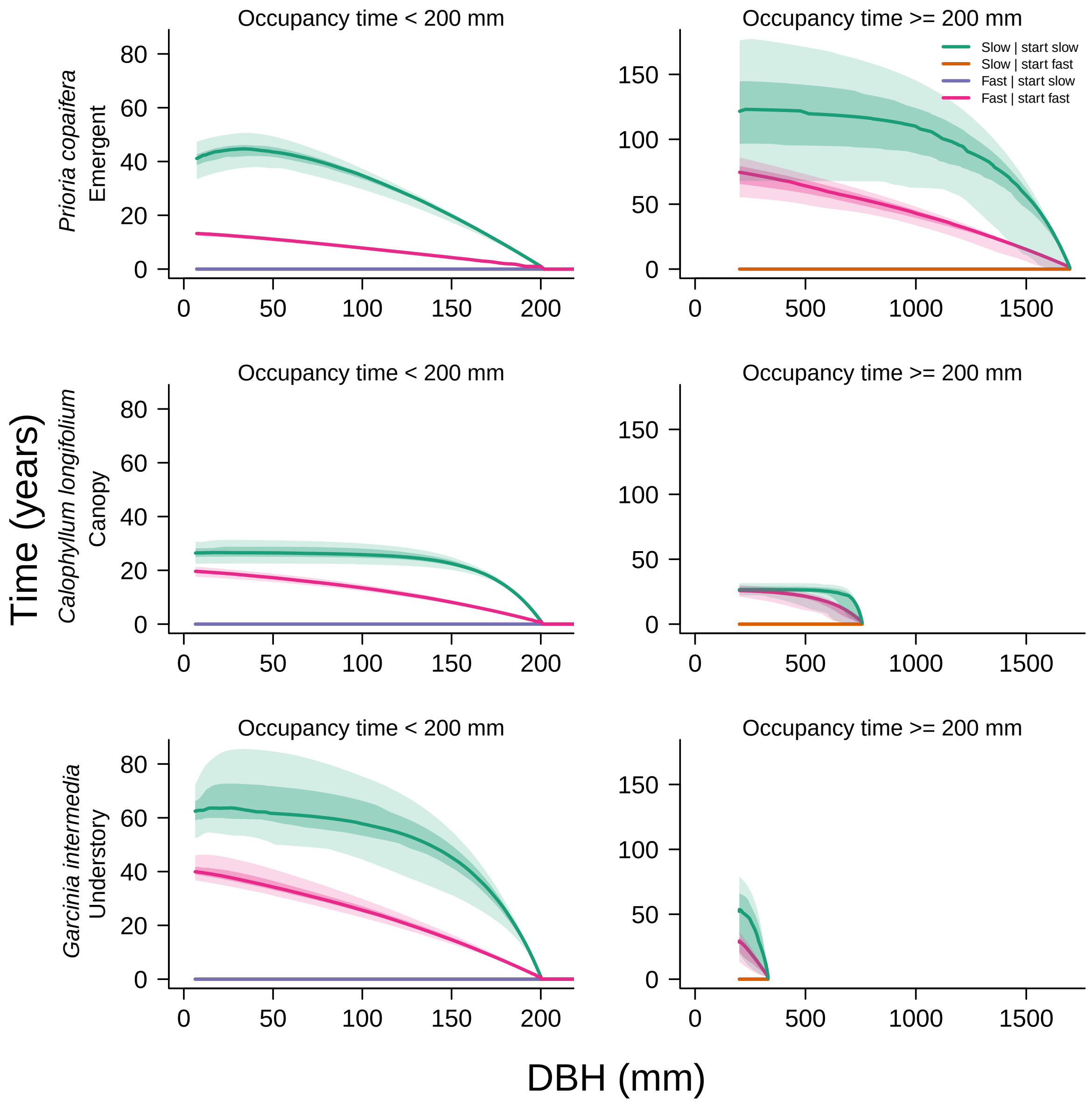

Figure A.3: Occupancy times for each BCI species in each growth distribution while below (left) and above (right) 200 mm DBH, conditional on starting growth distribution. Only *P. copaifera* was estimated to spend longer at sizes $\geq$ 200 mm DBH than < 200 mm DBH, a result of its later senescence and high asymptotic survival rates. Shaded polygons show 50th and 95th percentiles of uncertainty, propagated forward from posterior distributions of vital rate parameters.

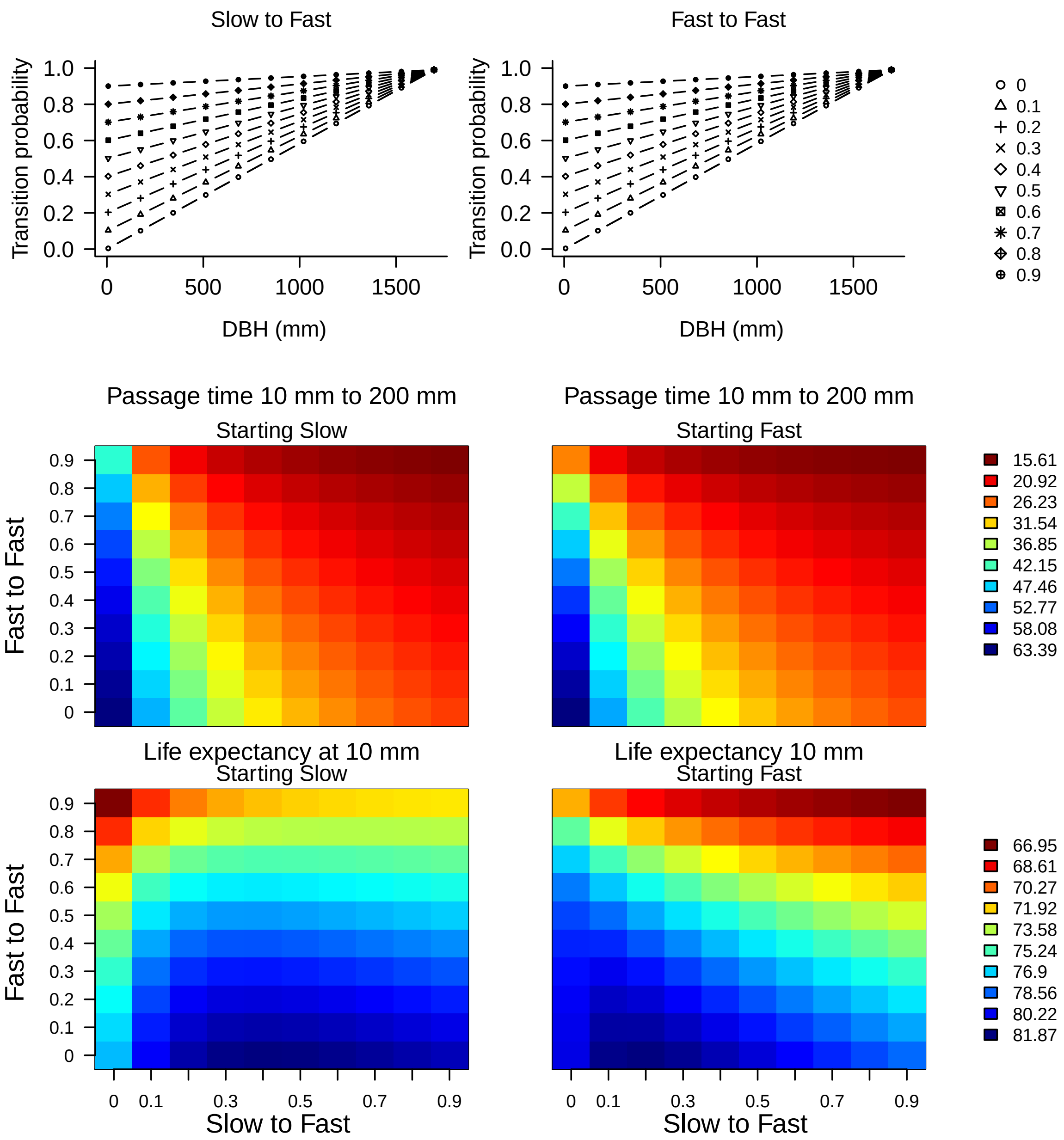

Figure A.4: Sensitivity of *P. copaifera* passage times from 10 to 200 mm DBH (middle row), and the life expectancy at 10 mm (bottom row), to the transition probabilities between growth distributions (top row). IPMs are constructed with size dependent transition probabilities between the slow and fast growth distributions. We kept the probability of slow to fast, and remaining fast, fixed at 0.99 at the largest size, and altered the transition probability at the smallest size, thus changing the gradient of the size-dependency. From each combination of transitions, we constructed IPMs and calculated passage times and life expectancies. Passage times are fastest when the probability of moving to and remaining in fast growth is high. With these combinations of transition probabilities, passage times but not life expectancies are more sensitive to fast to fast transitions than to the slow to fast transitions.

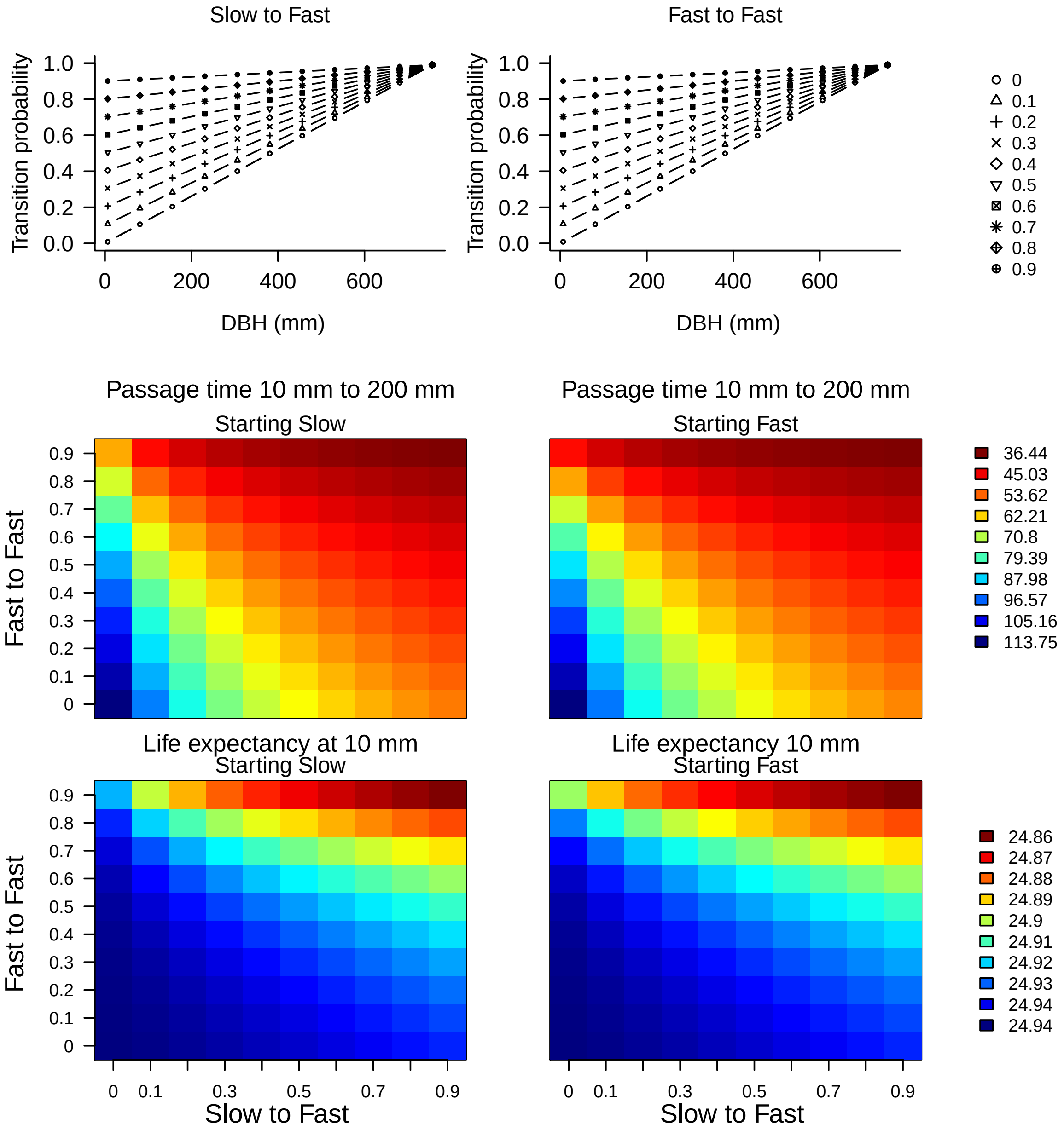

Figure A.5: Sensitivity of *C. longifolium* passage times from 10 to 200 mm DBH (middle row), and the life expectancy at 10 mm (bottom row), to the transition probabilities between growth distributions (top row). IPMs are constructed with size dependent transition probabilities between the slow and fast growth distributions. We kept the probability of slow to fast, and remaining fast, fixed at 0.99 at the largest size, and altered the transition probability at the smallest size, thus changing the gradient of the size-dependency. From each combination of transitions, we constructed IPMs and calculated passage times and life expectancies. Passage times are fastest when the probability of moving to and remaining in fast growth is high. With these combinations of transition probabilities, both passage times and life expectancies are more sensitive to slow to fast transitions than to the fast to slow transitions.

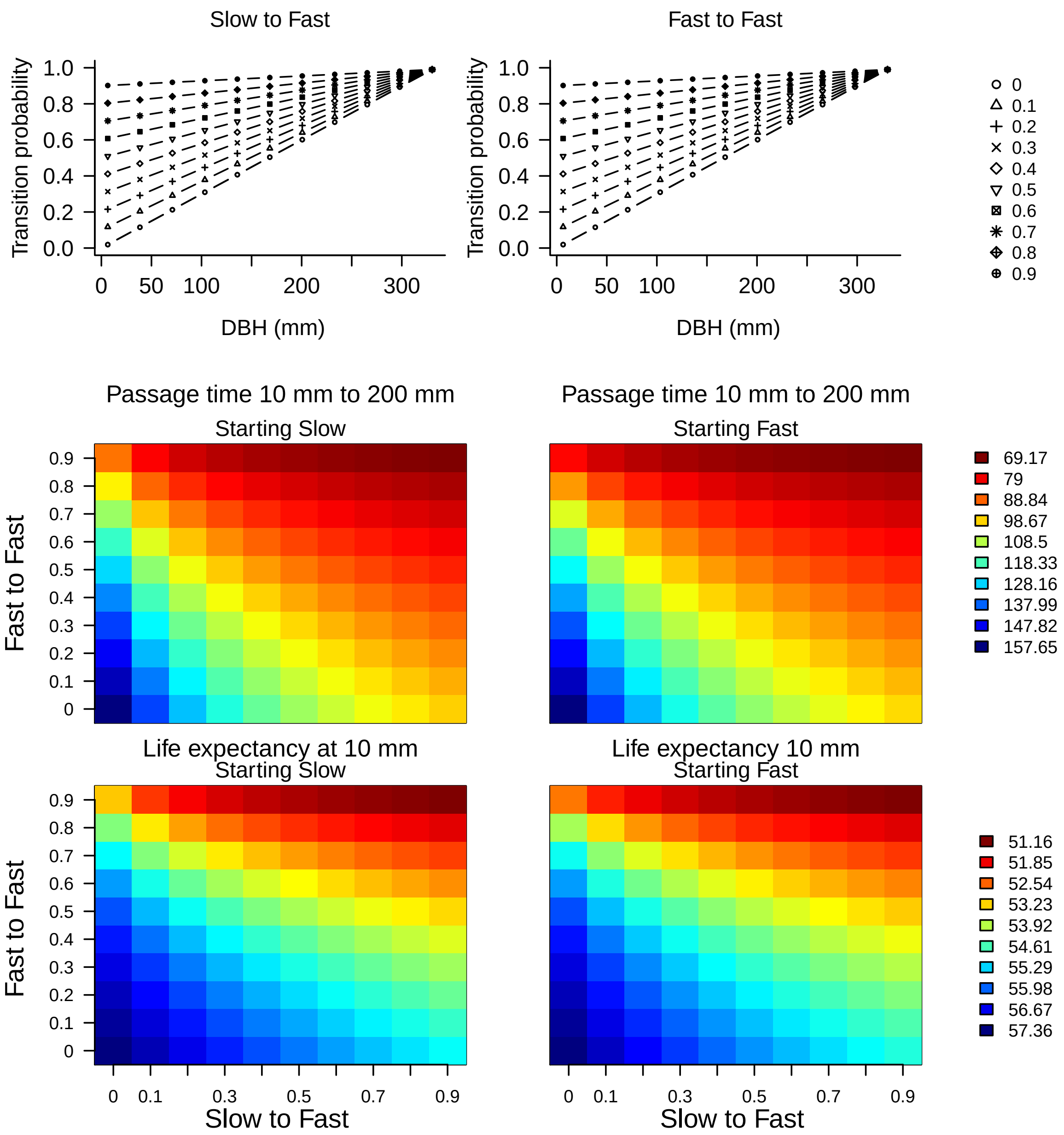

Figure A.6: Sensitivity of *G. intermedia* passage times from 10 to 200 mm DBH (middle row), and the life expectancy at 10 mm (bottom row), to the transition probabilities between growth distributions (top row). IPMs are constructed with size dependent transition probabilities between the slow and fast growth distributions. We kept the probability of slow to fast, and remaining fast, fixed at 0.99 at the largest size, and altered the transition probability at the smallest size, thus changing the gradient of the size-dependency. From each combination of transitions, we constructed IPMs and calculated passage times and life expectancies. Passage times are fastest when the probability of moving to and remaining in fast growth is high. With these combinations of transition probabilities, both passage times and life expectancies are more sensitive to slow to fast transitions than to the fast to slow transitions.

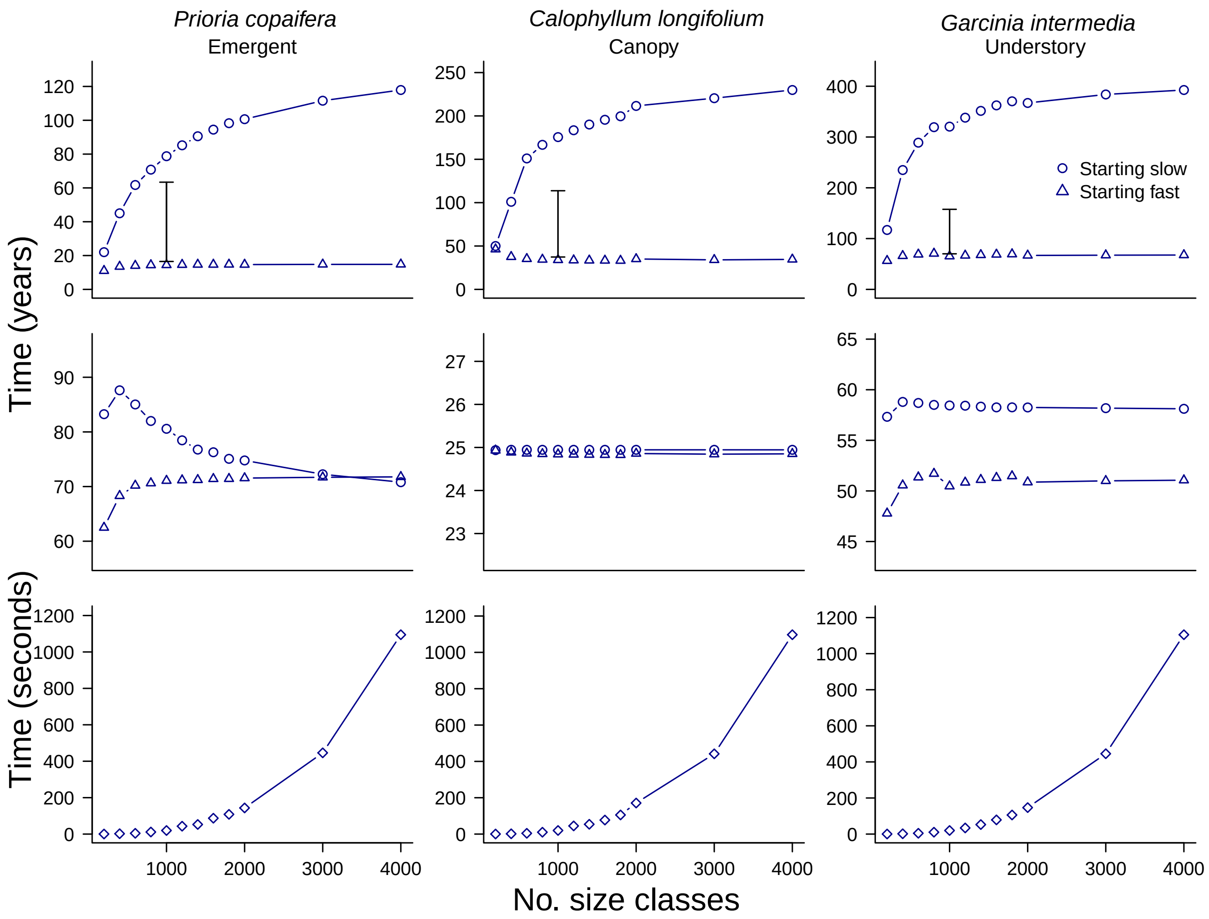

Figure A.7: Sensitivity of passage times from 10 to 200 mm DBH (top) and life expectancies (centre) at 10 mm DBH, to different IPM dimensions. Time taken to calculate passage times are shown in the bottom panels. Passage times in the slow growth distribution increased with increasing IPM dimensions. For reference, the range of passage times for slow growers from the sensitivity analysis to transitions between growth distributions is plotted. With non-zero transitions passage times for slow growers decrease. Life expectancies for slow growers decreased in *P. copaifera* (but remained almost constant in *C. longifolium* and *G. intermedia*). Time taken to calculate passage times increases non-linearly with increasing IPM size.

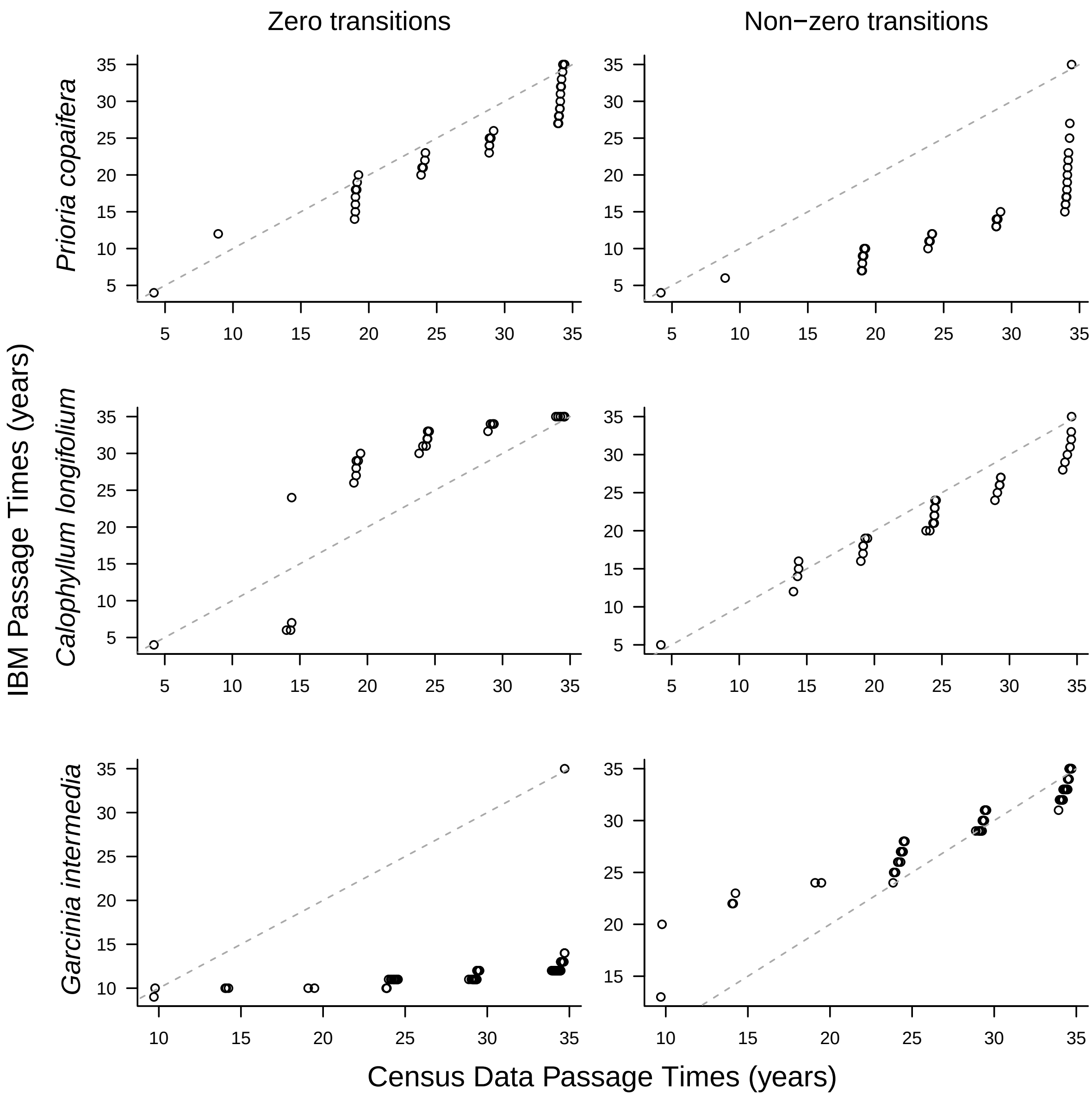

Figure A.8: QQ plots showing quantiles from the distribution of passage times estimated from census data and IBMs. IBMs either had zero transitions between growth distributions (left) or a simple linear function describing size dependent transitions (right). Only the first 34 years of the IBMs were used to make results comparable to the census data. Due to five year census intervals, the results from the census data are clustered around multiples of five. In the emergent species *P. copaifera*, results from the IBM with zero transitions more closely matched the distribution of passage times from the census data. In the other two species, allowing movement of individuals between slow and fast growth distributions resulted in a closer match of passage time distributions to census data.

10 Tables

V1		*Prioria copaifera*			*Calophyllum longifolium*			*Garcinia intermedia*	
	25th percentile	50th percentile	75th percentile	25th percentile	50th percentile	75th percentile	25th percentile	50th percentile	75th percentile
K	0.9902684	0.9917667	0.9930091	0.9593999	0.9614874	0.9635106	0.9864549	0.9874334	0.9884329
p1	-119.57979	-92.21558	-70.52457	-84.89838	-64.63998	-43.32463	-42.04992	-32.17602	-21.97308
r1	0.02718784	0.03365070	0.04173904	0.12314103	0.16544384	0.20724558	0.09524466	0.12024160	0.15826587
p2	1832.8341	2102.4999	2362.2350	728.4293	827.2177	929.4695	377.6428	431.3762	479.8177
r2	-0.07648835	-0.05171101	-0.02876837	-0.07754629	-0.05521623	-0.03368270	-0.08304766	-0.06447742	-0.04477094
surv.thresh	255.52965	261.52943	274.76203	99.10371	102.97067	109.65539	50.43154	52.63692	53.87716
Survival 10 mm	0.9567376	0.9607991	0.9645393	0.9583912	0.9606261	0.9627318	0.9783544	0.9798447	0.9813577
Survival rate 10 mm	7.343586e-04	9.823224e-04	1.332750e-03	4.958311e-08	4.397178e-06	7.906259e-05	5.927504e-04	8.237556e-04	1.121221e-03
Survival max DBH	0.9896996	0.9914815	0.9928285	0.9377072	0.9588848	0.9620970	0.9819123	0.9862023	0.9876635
Survival rate max DBH	-2.045056e-08	-1.643130e-14	0.000000e+00	-6.171700e-04	-9.051349e-06	-4.636016e-09	-2.336256e-04	-1.477030e-05	-1.942982e-07

Table A.1: Survival parameters. K = upper asymptote, p = inflection point, r = rate. 1 and 2 denote the curves for sizes below and above surv.thresh - the size threshold where the two curves meet. Survival 10 mm and Survival max DBH are the annual survival probability at 10 mm DBH and at the maximum observed DBH. Survival rate 10 mm and survival rate max DBH are the rate at which survival is increasing or decreasing at 10 mm DBH and the maximum observed DBH. Posterior distributions from all census intervals were combined in order to quantify uncertainty.

		alpha1	alpha2	beta1	beta2	DBH increment slow (mm/year)	DBH increment fast (mm/year)
Prioria copaifera	25th percentile	0.65	13.97	0.53	1.07	0.67	12.57
	50th percentile	0.66	15.73	0.55	1.21	0.69	12.80
	75th percentile	0.68	17.50	0.57	1.34	0.70	13.04
Calophyllum longifolium	25th percentile	0.97	7.25	1.60	1.49	0.41	4.79
	50th percentile	1.00	8.33	1.66	1.73	0.42	4.90
	75th percentile	1.03	9.55	1.73	1.98	0.43	5.03
Garcinia intermedia	25th percentile	1.12	10.81	2.47	4.36	0.33	2.53
	50th percentile	1.14	11.53	2.51	4.65	0.33	2.55
	75th percentile	1.15	12.30	2.55	4.98	0.34	2.57

Table A.2: Parameter values for the two distribution growth model. Alpha and beta refer to the shape and rate parameters for gamma distributions, with 1 and 2 corresponding to the slow and fast distributions respectively. The last two columns correspond to the expectation of growth in the slow and fast portions of the mixed distribution. The threshold dividing the slow and fast portions of the mixed distribution is calculated as he 0.95 quantile of observed increments for each species. Posterior distributions from all census intervals were combined in order to quantify uncertainty.

References

Caswell, H. (2012). Matrix models and sensitivity analysis of populations classified by age and stage: a vec-permutation matrix approach. *Theoretical Ecology*, 5, 404–417.

Easterling, M.R., Ellner, S.P. & Dixon, P.M. (2000). Size-specific sensitivity: Applying a new structured population model. *Ecology*, 81, 694–708.

Ireland, K.B., Moore, M.M., Fulé, P.Z., Zegler, T.J. & Keane, R.E. (2014). Slow lifelong growth predisposes *Populus tremuloides* trees to mortality. *Oecologia*, 175, 847–859.

Muller-Landau, H.C., Wright, S.J., Calderòn, O., Condit, R. & Hubbell, S.P. (2008). Interspecific variation in primary seed dispersal in a tropical forest. *Journal of Ecology*, 96, 653–667.

Pau, S., Wolkovich, E.M., Cook, B.I. *et al.* (2013). Clouds and temperature drive dynamic changes in tropical flower production. *Nature Clim. Change*, 3, 838–842.

R Core Team (2017). *R: A Language and Environment for Statistical Computing.* R Foundation for Statistical Computing, Vienna, Austria.

Rüger, N., Berger, U., Hubbell, S.P., Vieilledent, G. & Condit, R. (2011). Growth Strategies of Tropical Species: Disentangling Light and Size Effects. *PLoS One*, 6.

Sun, I.F., Chen, Y.Y., Hubbel, S.P., Wright, S.J. & Noor, N.S.M. (2007). Seed predation during general flowering events of varying magnitude in a malaysian rain forest. *Journal of Ecology*, 95, 818–827.

Uriarte, M., Canham, C., Thompson, J., Zimmerman, J. & Brokaw, N. (2005). Seedling recruitment in a hurricane-driven tropical forest: light limitation, density-dependence and the spatial distribution of parent trees. *Journal of Ecology*, 93, 291–304.

Williams, J.L., Miller, T.E.X. & Ellner, S.P. (2012). Avoiding unintentional eviction from integral projection models. *Ecology*, 93, 2008–2014.

Wright, S.J. & Calderón, O. (2006). Seasonal, el niño and longer term changes in flower and seed production in a moist tropical forest. *Ecology Letters*, 9, 35–44.

Zuidema, P.A., Jongejans, E., Chien, P.D., During, H.J. & Schieving, F. (2010). Integral Projection Models for trees: a new parameterization method and a validation of model output. *Journal of Ecology*, 98, 345–355.
