# Supplementary material for: Inferring forest fate from demographic data: from vital rates to population dynamic models
Source: Proc Biol Sci. 2018 Mar 7;285(1874):20172050. doi: 10.1098/rspb.2017.2050 (PMC5879618; doi:10.1098/rspb.2017.2050)
Supplement: Appendix 2: Theory for Vec-Permutation IPM analysis [file rspb20172050supp2.pdf]

Inferring forest fate from demographic data: from vital rates to population dynamic models: Appendix 2

Jessica Needham[1], Cory Merow[2], Chia-Hao Chang-Yang[1], Hal Caswell[3], and Sean M. McMahon[1]

[1]Smithsonian Institution Forest Global Earth Observatory, Smithsonian Environmental Research Center, 647 Contees Wharf Road, Edgewater, MD 21307-0028
[2]Ecology and Evolutionary Biology, Yale University, 165 Prospect St, New Haven, CT 06511-8934,
[3]Institute for Biodiversity and Ecosystem Dynamics (IBED), University of Amsterdam, Science Park 904, 1098 XH Amsterdam, The Netherlands

Contents

1 Introduction **1**

2 Notation **2**

3 Occupancy times **2**
3.1 Mixed distributions 3

4 Survivorship **3**
4.1 Mixed distributions 4

5 Longevity Statistics **4**

6 Passage Times **5**

1 Introduction

This appendix details the calculation of occupancy times, longevity statistics, and passage times for the model combining size and growth states. This requires extracting subsets of the population (e.g., in order to find expected occupancy

of a set of states) or combining mixtures of individuals in different states (e.g., a cohort that begins with individuals distributed among size or growth classes). An interested reader can find more details on these methods in Caswell (2013, 2014); Caswell *et al.* (In prep.); Roth & Caswell (2018).

2 Notation

Matrices are denoted by bold uppercase letters, e.g. $\mathbf{A}$. The entries of matrices are donated by corresponding lower case letters with subscripts for row and column indices, e.g. a_{ij} is the value in the ith row and j^{th} column of the matrix $\mathbf{A}$. $\mathbf{A}_{[i,.]}$ are the rows of $\mathbf{A}$, and $\mathbf{A}_{[.,j]}$ are the columns of $\mathbf{A}$. Vectors are denoted by bold lowercase letters and indexed with subscripts, e.g. v_i is the ith entry of the vector $\mathbf{v}$. Vec-permutation matrices for describing size-growth structured populations are denoted with a tilde, e.g. $\tilde{\mathbf{A}}$.

Other relevant points:

- $\mathbf{A}^{\mathsf{T}}$ is the transpose of the matrix $\mathbf{A}$.
- $\mathbf{A}^{-1}$ is the inverse of the matrix $\mathbf{A}$.
- $\mathbf{I}_i$ is an identity matrix of dimensions i.
- $\mathbf{A} \otimes \mathbf{B}$ is the Kronecker product of the matrices $\mathbf{A}$ and $\mathbf{B}$.
- $\mathbf{A} \circ \mathbf{B}$ is the Hadamard, or element-by-element, product of the matrices $\mathbf{A}$ and $\mathbf{B}$.
- S is the number of growth classes.
- G is the number of size classes.

3 Occupancy times

To find the expected occupancy time of each state (i.e. size and growth class combinations) we first define the fundamental matrix, $\tilde{\mathbf{N}}$, of $\tilde{\mathbf{P}}$.

$$\tilde{\mathbf{N}} = (\mathbf{I}_{SG} - \tilde{\mathbf{P}})^{-1} \tag{1}$$

This returns an SG x SG matrix, the entries of which give the expected occupancy time in state i, given starting in state j:

$$\tilde{\mathbf{v}}_{ij} = E(\text{occupancy of state } i | \text{ current state } j) \tag{2}$$

It is also possible to find the expected occupancy times in a set of states. If $\mathbf{c}_s$ is an S x 1 vector with 1s indicating the starting size classes of interest and 0s elsewhere then

$$\tilde{\mathbf{N}}(\mathbf{c}_S) = (\mathbf{I}_G \otimes \mathbf{c}_S^{\mathrm{T}})\tilde{\mathbf{N}} \tag{3}$$

where $\tilde{\mathbf{N}}(\mathbf{c}_S)$ is a G x SG matrix, in which columns are the starting states, arranged from left to right as sizes within growth classes, and rows correspond to growth classes. Thus, $\tilde{\mathbf{N}}(\mathbf{c}_S)_{ij}$ is the expected occupancy time in G_i summed over the size classes of interest, given starting in size-growth state j.

Likewise, the expected occupancy time in each size for the growth classes of interest is given by

$$\tilde{\mathbf{N}}(\mathbf{c}_G) = (\mathbf{I}_S \otimes \mathbf{c}_G^{\mathrm{T}})\tilde{\mathbf{N}} \tag{4}$$

where $\mathbf{c}_G$ is a G x 1 vector indicating growth classes of interest and $\tilde{\mathbf{N}}(\mathbf{c}_G)$ is an S x SG matrix in which $\tilde{\mathbf{N}}(\mathbf{c}_G)_{ij}$ is the expected occupancy time in S_i, summed over the growth classes of interest, given starting in size-growth state j.

Finally, combining the two, we get the expected occupancy time in each size and growth class of interest, $\mathbf{c}_S$ and $\mathbf{c}_G$, for each starting state.

$$\tilde{\mathbf{N}}(\mathbf{c}_S, \mathbf{c}_G) = (\mathbf{c}_G^{\mathrm{T}} \otimes \mathbf{c}_S^{\mathrm{T}})\tilde{\mathbf{N}} \tag{5}$$

3.1 Mixed distributions

To find the occupancy times for a cohort of individuals that are mixed among sizes and/or growth groups we first define the SG x 1 vector, $\tilde{\mathbf{n}}$ which holds the distribution of states in the population, arranged as sizes within growth groups e.g. for the case of G = 2

$$\tilde{\mathbf{n}}_t = \begin{pmatrix} n_{1,1} \\ n_{2,1} \\ \vdots \\ n_{S,1} \\ \hline n_{1,2} \\ n_{2,2} \\ \vdots \\ n_{S,2} \end{pmatrix}$$

The occupancy times of this cohort in each state is found by multiplying $\tilde{\mathbf{n}}$ by the fundamental matrix $\tilde{\mathbf{N}}$

$$\tilde{\mathbf{N}}(\tilde{\mathbf{n}}) = \tilde{\mathbf{N}}\tilde{\mathbf{n}} \tag{6}$$

4 Survivorship

The survivorship of an initial cohort after x years is given by

$$\tilde{\mathbf{l}}(x) = (1_{SG}^{\mathrm{T}} \tilde{\mathbf{P}}^{x})^{\mathrm{T}} \tag{7}$$

where $\tilde{\mathbf{l}}$ is an SG x 1 vector in which the ith entry is the survivors to age x of a cohort starting in state i.

4.1 Mixed distributions

To find survivorship for a cohort with a mixed distribution of starting states we define $\mathbf{n}$, a vector with the size distribution of the cohort (not separated by growth group). The survivorship of all sizes within each growth group after x years is given by:

$$\tilde{\mathbf{l}}(x|\mathbf{n}) = (\mathbf{I}_G \otimes \mathbf{n}_s)^{\mathrm{T}} \tilde{\mathbf{l}} \tag{8}$$

5 Longevity Statistics

The mean, variance and skew of longevity can be calculated from the fundamental matrix $\tilde{\mathbf{N}}$.

Since $\tilde{\mathbf{N}}$ gives the expected number of visits to each state i, from state j, the column sums of $\tilde{\mathbf{N}}$ give the mean longevity of each starting state j.

$$\tilde{\eta}_1^{\mathrm{T}} = 1_{SG}^{\mathrm{T}} \tilde{\mathbf{N}} \tag{9}$$

The second moment of longevity is given by

$$\tilde{\eta}_2^{\mathrm{T}} = \tilde{\eta}_1^{\mathrm{T}} (2\tilde{\mathbf{N}} - I_{SG}) \tag{10}$$

which leads to the variance in longevity

$$1_{SG}^{\mathrm{T}} (2\tilde{\mathbf{N}}^{\mathbf{2}} - \tilde{\mathbf{N}}) - 1_{SG}^{\mathrm{T}} \tilde{\mathbf{N}} \circ 1_{SG}^{\mathrm{T}} \tilde{\mathbf{N}} \tag{11}$$

The third moment of longevity, which measures the skewness in the distribution of longevities, is given by

$$\tilde{\eta}_3^{\mathrm{T}} = \tilde{\eta}_1^{\mathrm{T}} (6\tilde{\mathbf{N}}^{\mathbf{2}} - 6\tilde{\mathbf{N}} + I_{SG}) \tag{12}$$

Finally, we can compute the full probability distribution of longevities for each state as

$$p(\eta = n| \text{ start in } i) = [1_{SG}^{\mathrm{T}} (I - \mathbf{P}) \mathbf{P}^{n-1}]^{\mathrm{T}} \tag{13}$$

6 Passage Times

To calculate passage times to states of interest, $\Re$, we create a Markov chain in which states of interest are absorbing states, and transition probabilities into those states are conditional on survival to those states. The transition matrix of the conditional Markov chain is given by

$$\tilde{\mathbf{P}} = \left(\begin{array}{c|c} \tilde{\mathbf{P}}' & 0 \\ \hline \tilde{\mathbf{M}}' & 1 \end{array}\right)$$

$\tilde{\mathbf{P}}'$ is equal to $\tilde{\mathbf{P}}$ but with 0s in the rows and columns of $\Re$, since transitions into these states means absorption has already occured.

$\tilde{\mathbf{M}}'$ is a 2 x SG matrix. The first row holds the probabilities of death before reaching the absorbing states of interest, i.e. $1 - \sum \tilde{\mathbf{P}}_{[.,j]}$, for $j \neq \Re$ and 0 for $j = \Re$, since individuals in $\Re$ have already been absorbed. The second row of $\tilde{\mathbf{M}}'$ holds the probability of reaching $\Re$ before death, i.e. $\sum \tilde{\mathbf{P}}_{[\Re,j]}$ for $j \neq \Re$ and 1 for $j = \Re$, since individuals in $\Re$ have already been absorbed.

The conditional fundamental matrix $\tilde{\mathbf{N}}^c$ is given by

$$\tilde{\mathbf{N}}^c = \mathbf{D}^{-1}\tilde{\mathbf{N}}\mathbf{D} \tag{14}$$

where, with diag($\mathbf{b}_{k.}$) denoting the kth row of $\mathbf{B}$,

$$\mathbf{D} = \text{diag}(\mathbf{b}_{k.}) = \begin{pmatrix} b_{k1} & & \\ & b_{k2} & \\ & & \ddots \end{pmatrix} \tag{15}$$

and

$$\mathbf{B} = \mathbf{M}\tilde{\mathbf{N}} \tag{16}$$

and

$$\tilde{\mathbf{N}} = (\mathbf{I}_{SG} - \tilde{\mathbf{P}})^{-1} \tag{17}$$

$\tilde{\mathbf{N}}^{\mathbf{c}}$ can be analysed like any other fundamental matrix. Thus, the time to abosorbtion in the states of interest (passage times), $\Re$ are given by the column sums of $\tilde{\mathbf{N}}^{\mathbf{c}}$, while the variance and skew of passage times can be calculated from the second and third moments.

References

Caswell, H. (2013). Sensitivity analysis of discrete markov chains via matrix calculus. *Linear Algebra and its Applications*, 438, 1727 – 1745. 16th ILAS Conference Proceedings, Pisa 2010.

Caswell, H. (2014). A matrix approach to the statistics of longevity in heterogeneous frailty models. *Demographic Research*, 31, 553–592.

Caswell, H., de Vries, L., Hartemink, N., Roth, G. & van Daalen, S. (In prep.). Age x stage-classified demography: a comprehensive approach.

Roth, G. & Caswell, H. (2018). Occupancy time in sets of states for demographic models. *Theoretical Population Biology*, 120, 62 – 77.
