# Supplementary material for: Inferring forest fate from demographic data: from vital rates to population dynamic models
Source: Proc Biol Sci. 2018 Mar 7;285(1874):20172050. doi: 10.1098/rspb.2017.2050 (PMC5879618; doi:10.1098/rspb.2017.2050)
Supplement: Appendix 3: R scripts for the complete workflow [file rspb20172050supp3.pdf]

Inferring forest fate from demographic data: from vital rates to population dynamic models: Appendix 3

Needham, J., Merow, C., Chang-Yang, C-H., Caswell, H., and McMahon, S.M.

These scripts have been written as if the user has a treeIPM directory containing a Source folder for R scripts, a Data folder for data, and Outputs and Figures folders for outputs generated by the code. Scripts run as if the working directory is set to the treeIPM directory. In all the scripts below involving random number generation, we set the seed to make our results reproducible.

Data Preparation

```
######################################################################
#### TIDYING UP DATA FROM MULTIPLE CENSUSES ##########################
# APPENDIX VERSION #

# In this script we load census data from 1995 to 2015,
# chose a species, and correct measurement errors
######################################################################
rm(list = ls())  # clear the work space
# Set working directory to the treeIPM folder
#setwd('SET YOUR PATH HERE/treeIPM')
set.seed(1)  # set the seed to make results reproducible

###########################
### LIBRARIES ###
###########################
library(plyr)
library(foreach)
library(doParallel)
library(SpatialTools)
library(stringi)
library(stringr)

#############################
### FUNCTIONS ###
#############################
source('Source/Data_processing_functions.R')
# number of cores to use for parallel processing
nproc <- detectCores()-2
registerDoParallel(nproc)

##############################
### DATA ###
##############################
# load in censuses - here 1995 - 2015
censuses <- seq(4,8,1)
n.census <- length(censuses)
site.name <- 'bci'
dbh.factor <- 1  # needs to be 10 if data is in cm
```

```
# load each census
for (i in censuses){
  load(paste('Data/', site.name,
             ".stem", i, ".rdata", sep = ""))
}

# put data from each census in a list
cns <- vector('list', n.census)

for(i in 1:n.census){
  cns[[i]] = eval(as.name(paste(site.name,
                                ".stem",censuses[i], sep="")))
}

### choose the species
sp.name <- 'calolo'
cns <- lapply(cns, function(x)
  x[x$sp == sp.name, ])

# rearrange columns and only keep tags, species,
# coordinates, date
cnsdata = list()

for (i in 1:n.census){
  cnsdata[[i]] <- cns[[i]][,c('treeID', 'stemID', 'tag', 'StemTag',
                              "sp", "gx", "gy", "dbh",
                              "date")]
}

# convert all dbhs to mm units
for(i in 1:n.census){
  cnsdata[[i]]$dbh <-cnsdata[[i]]$dbh * dbh.factor
}

# this appends the census number to time-dependent variables in each census
# (i.e. dbh becomes dbh.1)
for(i in 1:n.census){
  colnames(cnsdata[[i]])[8:ncol(cnsdata[[i]])] <-
    paste(colnames(cnsdata[[i]])[8:ncol(cnsdata[[i]])],".",i, sep="")
}

# Combine list items into a data frame
# select static information from first census
indv  <- cnsdata[[1]][,1:7]
temp2 <- lapply(cnsdata,"[", -c(1:7))
# bind dynamic data to static data
tmp <- cbind(indv, do.call(cbind ,(temp2)))
rm(temp2)
rm(indv)

# years that the censuses were conducteded
years <- c('1995', '2000', '2005', '2010', '2015')
```

```
# convert dates to time in years since first measurment
start.d <- min(tmp$date.1, na.rm=TRUE)
for(i in 1:n.census){
  tmp[ ,grep(paste('date', i, sep = '.'), colnames(tmp))] <-
    (tmp[ ,grep(paste('date', i, sep ='.'), colnames(tmp))] - start.d)/365.25
}

# make a survival column - for the stem data this needs to be based on
# dbh and NOT on status - since status column refers to the whole tree

# survival based on NAs in sizes
size.mat <- tmp[ ,grep('dbh', colnames(tmp))]

for(i in 1:(n.census-1)){
  S <- ifelse(is.na(size.mat[ ,i+1]) &
                !is.na(size.mat[ ,i]), 0, NA)
  S <-  ifelse(!is.na(size.mat[ ,i+1]) &
                 !is.na(size.mat[ ,i]), 1,
               S)
  tmp[ ,paste0('Surv.', i)] <- S
}

# check it worked (should all be 0)
surv.mat <- tmp[ ,grep('Surv', colnames(tmp))]
for(i in 1:(n.census-1)){
  k <- length(which(is.na(surv.mat[ ,i]) &
                      !is.na(size.mat[ ,i])))
  print(k)
}

# get rid of trees with no measurement in any census
sizes <- tmp[ ,grep('dbh', colnames(tmp))]
all.na <- which(apply(sizes, 1, function(x) all(is.na(x))))
if(length(all.na)>0){
  tmp <- tmp[-all.na, ]
}

data.mat <- tmp

# tidy the workspace
remove(cns)
remove(cnsdata)
remove(tmp)

##########################
# GROWTH CORRECTION #

# for each species get rid of those with individuals more than twice the
# 99th quantile of size.
data.mat <- remove.too.big(data.mat)

# make an increment column
sizes <- as.matrix(data.mat[ ,grep('dbh', colnames(data.mat))])
```

```r
incrs <- t(apply(sizes, 1, diff))

if(n.census == 2){
  incrs <- t(incrs)
}
# name them
colnames(incrs) <- paste('incr', seq(n.census -1), sep = '.')

# get time in years between censuses
times <- data.mat[ ,grep('date', colnames(data.mat))]
times <- t(apply(times, 1, diff))

if(n.census == 2){
  times <- t(times)
}
colnames(times) <- paste('time', seq(n.census-1), sep = '.')

# survival
survival <- as.matrix(data.mat[ ,grep('Surv', colnames(data.mat))],
                      ncol = n.census -1)

# coordinates
gx <- as.numeric(data.mat$gx)
gy <- as.numeric(data.mat$gy)
# make it a matrix - much quicker to process
sp.id <- 1  # make species a numeric
# make the tags numeric
data.mat[ ,grep('tag', colnames(data.mat), ignore.case = TRUE)] <-
  apply(data.mat[ ,grep('tag', colnames(data.mat), ignore.case = TRUE)],
        2, function(x) as.numeric(x))

data.mat <- as.matrix(cbind(as.matrix(data.mat[ ,1:4]),
                            sp.id, gx, gy, sizes, survival,
                            incrs, times))

#  returns true if the stem shrank more than 25% of original size
# or grew more than 75 mm in a single year
# correct those obvious outliers - make them the mean of measurements either side
# or if they are first or last make them second/penultimate -/+ growth from closest
# census - only works when there are >2 measurements
if(length(grep('dbh', colnames(data.mat))) > 2){
  errors <- which(apply(data.mat, 1, find.errors) == TRUE)
  if(length(errors) > 1){
    data.mat[errors, ] <- t(apply(data.mat[errors, ], 1, correct.errors))
  }else{
    if(length(errors) == 1){
      data.mat[errors, ] <- t(correct.errors(data.mat[errors,]))
    }
  }

  # get new increments
  sizes <- data.mat[ ,grep('dbh', colnames(data.mat))]
  incrs.ll <- t(apply(sizes, 1, diff))
```

```
  data.mat[ ,grep('incr', colnames(data.mat))] <- incrs.ll
}

#  Remove stems dead in all censuses
bb <- data.mat[ ,grep('dbh', colnames(data.mat))]
data.mat <- data.mat[which(apply(bb, 1,
                                 function(x) all(is.na(x)))
                           == FALSE), ]

# - propose new sizes so that size monotonically increases
reps <- 1  # keep this at one - otherwise the median becomes
# inflated from  'large' corrections
give.up <- 10000 #  attempts at getting positive increments

dbhs <- data.mat[ ,grep('dbh',
                        colnames(data.mat))]  # matrix of sizes
# matrix of times
times <- as.matrix(data.mat[ ,grep('time',
                                   colnames(data.mat))], ncol = n.census-1)
# split dbhs and times into chuncks for parallel processing
chunk.size <- dim(dbhs)[1]/nproc
d <- seq(dim(dbhs)[1])
e <- split(d, ceiling(seq_along(d)/chunk.size))
if(length(e) > nproc){
  e[[nproc]] <- c(e[[nproc]], e[[length(e)]])
  e[[nproc +1]] <- NULL
}
dbh.list <- lapply(e, function(x, y) y[x, ], y = dbhs)
times.list <- lapply(e, function(x, y) y[x, ], y = times)

out <- foreach(proc = 1:nproc)%dopar% {
  set.seed(1)
  # growth correction code
  resampled.dbhs <-
    replicate(reps,
              correct.dbh(dbh.list[[proc]],
                          times = times.list[[proc]],
                          give.up = give.up))

  sampled <- unlist(resampled.dbhs[2,])
  dd <- resampled.dbhs[1, ]
  ee <- array(unlist(dd),
              dim = c(nrow(dd[[1]]), ncol(dd[[1]]), length(dd)))
  ff <- aperm(ee)

  # adjust the increments to match the new sizes
  med.corrections <- t(apply(ff, 3,
                             function(x) apply(x, 2, median)))

  return(list(med.correct = med.corrections,
              still.neg = sampled))
}
```

```
# number of trees that the algorithm failed on - these trees
# had increments sampled from positive increments of other trees
sampled <- unlist(lapply(out, '[[', 2))
print(sprintf('total sampled: %d', sampled))

# corrected sizes
med.corrections <- do.call(rbind, lapply(out, '[[', 1))

# replace the sizes with these corrected ones
data.mat[ ,grep('dbh', colnames(data.mat))] <- med.corrections
# make sure they are all still monotonically increasing
incrs.lll <- t(apply(med.corrections, 1, diff))

# replace the increment columns with the new positive increments
data.mat[ ,grep('incr', colnames(data.mat))] <- incrs.lll

#### load and format the species names
load(sprintf('Data/%s_spptable.rdata',
             site.name))
tmp <- eval(parse(text = sprintf('%s.spptable', tolower(site.name))))
# match the species codes from the chosen species with the codes from
# species tables
tmp <- as.data.frame(tmp)
sps <- match(sp.name, tmp$sp)
sp.latin <- cbind(tmp$Latin[sps], tmp$sp[sps])

# save the output
data.ls <- list(data.mat = data.mat,
                sp.name = sp.name,
                sp.latin = sp.latin,
                years = years)
saveRDS(data.ls,
        file = sprintf('Output/%s_%s.RData', site.name, sp.name))
```

Growth MCMC

```
##########################################
### GROWTH WRAPPER - LOADS STAN MCMC ###
##########################################
rm(list = ls())
set.seed(1)
##########################
### LIBRARIES ###
##########################
library(rstan)
#library(shinystan)
library(MASS)

################################
### DATA ###
################################
site <- 'bci'
```

```
sp.name <- 'calolo'
data.ls <- readRDS(file = sprintf('Output/%s_%s.RData', site, sp.name))
data.mat <- data.ls$data.mat
sp.latin <- data.ls$sp.latin[ ,1]
years <- data.ls$years

n.census <- length(grep('dbh', colnames(data.mat)))
n.intervals <- n.census - 1
n.iter <- 2500  # number of iterations in the MCMC
quant <- 0.95  # proportion of the population in slow growth
G <- 2  # number of growth distributions
# dummy data so we can compile model outside of the
# foreach loop
N <- 100
incr <- rnorm(N, 1, 0.2)
incr.groups <- rbinom(N, 1, 0.05)

incr.data <- list(N = length(incr),
                  incr = incr,
                  groups = incr.groups)

# compile the model -
# this may take about 40-60 secs
growth.model <- stan(file = 'Source/gamma_growth.stan',
                     data = incr.data, chains = 0, verbose = FALSE)

# structures to hold the MCMC outputs
g.params <- vector('list', 3)
# first element is matrix of median parameter values
# in each census interval
g.params[[1]] <- matrix(NA, nrow = n.intervals, ncol = 4)
# second element is a list which holds the MCMC chains from
# each census interval
g.params[[2]] <- vector('list', n.intervals)
# to hold the increment threshold each census interval
g.params[[3]] <- rep(NA, n.intervals)

# for each census...
for(census in 1:n.intervals){
  size <- data.mat[ ,grep('dbh', colnames(data.mat))[(census)]]  # size start of census
  incr <- data.mat[ ,grep('incr', colnames(data.mat))[(census)]]  # incr
  time <- data.mat[ ,grep('time', colnames(data.mat))[census]]  # time
  treeID <- data.mat[ ,grep('treeID', colnames(data.mat))]
  # for each tree that lived get the largest stem that survived

  # remove stems dead/not recruited in either of the focal census years
  dead <- which(is.na(incr))
  size <- size[-dead]
  incr <- incr[-dead]
  time <- time[-dead]
  treeID <- treeID[-dead]

  # get increments of individual trees not stems
```

```
if(length(treeID) > 0){

  # index of largest stem per tree
  max.size <- tapply(size, treeID, which.max)
  # corresponding to rows...
  size <- tapply(size, treeID, max)  # max size of each tree

  # increment corresponding to largest stem of each tree
  incr <- mapply(function(x,y) x[y],
                 x = split(incr, treeID),
                 y = max.size)
  # time between censuses for largest stem per tree
  time <- mapply(function(x,y) x[y],
                 x = split(time, treeID),
                 y = max.size)
}

# for trees with no time measurements give them
# a random time from another stem
time[is.na(time)] <- sample(time[!is.na(time)],
                            length(which(is.na(time))))

# if there are less than 100 individuals in a census interval then
# put NAs and move on
if(length(size) < 100){
  next()
}

# MAKE INCREMENT ANNUAL #
incr <- as.numeric(incr/time)
g.params[[3]][census] <- quantile(incr, prob = quant)
###################################################
### MCMC ###

# INCREMENT GROUP
quant.incr <- g.params[[3]][census]
# divide the data into increment groups based on these increment thresholds
incr.groups <- cut(incr,
                   breaks = c(0, quant.incr, Inf),
                   labels = FALSE)

# put the data into a list object
incr.data <- list(N = length(incr),
                  incr = incr,
                  groups = incr.groups)

# get estimates of parameters to use as starting values in the STAN
# model - this helps speed things up
sp.1 <- as.numeric(fitdistr(incr[incr.groups == 1], 'gamma')$estimate)
sp.2 <- as.numeric(fitdistr(incr[incr.groups == 2], 'gamma')$estimate)

start.params <- list(alpha1 = sp.1[1], alpha2 = sp.2[1],
                     beta1 = sp.1[2], beta2 = sp.2[2])
```

```
  # parameter sampling
  growth.fit <- stan(fit = growth.model, data = incr.data, verbose = FALSE,
                     iter = n.iter, chains = 3,
                     init = list(start.params, start.params, start.params),
                     control = list(adapt_delta = 0.99,
                                    max_treedepth = 13))

  # check the MCMC results
  #summary(growth.fit)

  # extract the parameters
  # median values from each census interval go into a matrix
  growth.mcmc <- unlist(lapply(extract(growth.fit, permuted = TRUE), median))
  g.params[[1]][census, 1:G] <- growth.mcmc[1:2]
  g.params[[1]][census, (G+1):(G*2)] <- growth.mcmc[3:4]
  colnames(g.params[[1]]) <- names(growth.mcmc)[1:4]
  g.params[[2]][[census]] <- do.call(cbind, extract(growth.fit))

  print(census)

}
# save the whole output
save(g.params, file = sprintf('Output/%s_growthps_%s.RData',
                              site, sp.name))

# Make parameter tables #
intervals <- na.omit(expand.grid(years, years)[(seq(5)*(n.census+1)), ])
intervals <- paste(intervals[,1], intervals[,2], sep = '-')

gr.ps <- g.params[[1]]
gr.ps <- as.data.frame(gr.ps)
gr.ps <- cbind(intervals, gr.ps, stringsAsFactors = FALSE)

gr.ps <- cbind(c(sp.latin, rep(' ', nrow(gr.ps)-1)), gr.ps,
               stringsAsFactors = FALSE)

colnames(gr.ps) <- c('Species', 'Census', 'Alpha1', 'Alpha2', 'Beta1', 'Beta2')

rownames(gr.ps) <- NULL

# to make latex style tables
library(xtable)
gr.tab <- print(xtable(gr.ps), include.rownames = FALSE,
                digits = 3)

# plot the chains to ensure they are well mixed
par(mfrow = c(4,4))
lapply(g.params[[2]],
       function(x){
         apply(x[ ,1:4], 2, plot, type = 'l')
       })
```

Survival MCMC

```
############################################
### SURVIVAL WRAPPER - LOADS STAN MCMC ###
############################################
rm(list = ls())
set.seed(1)

###########################
### LIBRARIES ###
###########################
library(rstan)
#library(shinystan)

################################
### DATA ###
################################
site <- 'bci'
sp.name <- 'calolo'

data.ls <- readRDS(file = sprintf('Output/%s_%s.RData', site, sp.name))
data.mat <- data.ls$data.mat
sp.latin <- data.ls$sp.latin[ ,1]
years <- data.ls$years

n.census <- length(grep('dbh', colnames(data.mat)))
n.intervals <- n.census - 1
n.iter <- 2500  # number of iterations for the MCMC

# Run the MCMC -
n.census <- length(grep('dbh', colnames(data.mat)))
n.intervals <- n.census - 1

# here we just generate some fake data in order to pass it to stan()
# function so that we can compile the stan model outside of the
# foreach loop
N <- 100
size <- rnorm(N, 50, 5)
surv <- rbinom(N, 1, 0.1)
time <- rep(5, N)
thresh <- 30
lowr2 <- 20
upr2 <- 200

# make the data list to run stan model
surv.data <- list(N = length(size),
                  dbh = size,
                  surv = surv,
                  time = time,
                  thresh = thresh,
                  lowr2 = lowr2,
                  upr2 = upr2)
```

```
# compile the model (it may take 40-60 secs)
surv.model <- stan(file = 'Source/surv.stan',
                   data = surv.data, chains = 0)

# to hold outputs
s.params <- vector('list', 2)
# first element is a matrix of median parameter values per
# census interval
s.params[[1]] <- matrix(NA, nrow = n.intervals, ncol = 7)
# second element is a list of complete MCMC chains
s.params[[2]] <- vector('list', n.intervals)

# get time in years between censuses
times <- data.mat[ ,grep('time', colnames(data.mat))]

if(n.intervals == 1){
  times <- matrix(times, ncol = 1)
}

# now for each interval run the survival model
for(int in 1:(n.intervals)){

  # get vectors of size survival and time
  # because we are working with stem data need to make sure that
  # all stems of a tree die before we call it dead
  size <- data.mat[ ,grep('dbh', colnames(data.mat))[int]]
  surv <- data.mat[ ,grep('surv', colnames(data.mat),
                          ignore.case = TRUE)[(int)]]
  time.vec <- times[ ,int]
  treeID <- data.mat[ ,grep('treeID', colnames(data.mat))]

  # only keep stems that are alive in the first census
  no.sizes <- which(is.na(size))

  if(length(no.sizes)>0){
    size <- size[-no.sizes]
    surv <- surv[-no.sizes]
    time.vec <- time.vec[-no.sizes]
    treeID <- treeID[-no.sizes]
  }

  # for those sites with treeIDs
  if(length(treeID) > 0){

    # whole trees alive or dead?
    # vector length of no. trees with alive dead for whole tree
    tree.survs <- tapply(surv, treeID,
                         mean, na.rm = TRUE)
    # only those which are all dead (i.e. a 0) get called dead
    tree.survs <- ifelse(tree.survs == 0, 0, 1)

    # stem surv variable = whole tree level
    stem.tree.surv <- tree.survs[match(treeID, names(tree.survs))]
```

```
    # for trees that lived get largest stem that lived
    # for trees that died largest stem before death
    tree.size <- rep(NA, length(tree.survs))
    surv.mat <- cbind(treeID, size, stem.tree.surv, surv)

    trees.lived <- matrix(surv.mat[surv.mat[ ,3] == 1, ],
                          ncol = 4)

    # stems that died get an NA for size ...
    trees.lived[,2] <- ifelse(trees.lived[,4] == 0, NA, trees.lived[,2])
    # ... so that the max size for the tree is the largest stem that lived
    tree.size[tree.survs == 1] <- tapply(trees.lived[,2],
                                         trees.lived[,1], max, na.rm = TRUE)
    # largest stem of trees that died
    trees.died <- matrix(surv.mat[surv.mat[,3] == 0, ], ncol = 4)
    tree.size[tree.survs == 0] <- tapply(trees.died[,2],
                                         trees.died[,1], max, na.rm = TRUE)
    # make the tree level time vector
    tree.time <- unlist(tapply(time.vec, treeID,
                               function(x) names(which.max(table(x)))))
    # if a stem has no time measurement then give it the time of another tree
    tree.time.ll <- rep(NA, length(tree.size))
    tree.time.ll[match(names(tree.time), unique(treeID))] <- as.numeric(tree.time)
    tree.time.ll[is.na(tree.time.ll)] <- sample(tree.time.ll, 1)

    size <- tree.size
    surv <- tree.survs
    time <- tree.time.ll

    rm(tree.size, tree.survs, tree.time, tree.time.ll,
       surv.mat, trees.lived, trees.died)

  }else{
    time <- time.vec
  }
  time <- ifelse(is.na(time), 5,
                 time)

  # if there are less than 100 individuals in a census interval then
  # put NAs and move on
  if(length(size) < 100){
    next()
  }

  max.size <- max(size, na.rm = TRUE)

  # priors for the rate of senescence parameter
  lowr2 <- ifelse(max.size < 200, -0.5, -0.1)
  upr2 <- ifelse(max.size < 200, -0.01, -0.00001)

  thresh <- ifelse(max.size < 50, median(size),
                   max.size*0.2)
```

```
  # make the data list to run stan model
  surv.data <- list(N = length(size),
                    dbh = size,
                    surv = surv,
                    time = time,
                    thresh = thresh,
                    lowr2 = lowr2,
                    upr2 = upr2)


  # run the MCMC
  surv.fit <- stan(fit = surv.model, data = surv.data, iter = n.iter,
                   chains = 3, control = list(adapt_delta = 0.98))

  # extract the parameters
  surv.mcmc <- lapply(extract(surv.fit), median)
  surv.chains <- do.call(cbind, extract(surv.fit))

  s.params[[2]][[int]] <- surv.chains  # save whole output

  s.params[[1]][int, 1:3] <- unlist(surv.mcmc)[c(1,2,3)]
  s.params[[1]][int, 4:6] <- unlist(surv.mcmc)[c(1,4,5)]
  s.params[[1]][int, 7] <- thresh

}

colnames(s.params[[1]]) <- c('K', 'p1', 'r1',
                             'K', 'p2', 'r2', 'thresh')
save(s.params,
     file = sprintf('Output/%s_survps_%s.RData',
                    site, sp.name))

# make parameter tables
intervals <- na.omit(expand.grid(years, years)[(seq(5)*(n.census+1)), ])
intervals <- paste(intervals[,1], intervals[,2], sep = '-')

surv.ps <- s.params[[1]][ ,c(1,2,3,5,6,7)]
surv.ps <- as.data.frame(surv.ps)
surv.ps <- cbind(intervals, surv.ps, stringsAsFactors = FALSE)
surv.ps <- cbind(c(sp.latin, rep(' ', nrow(surv.ps)-1)), surv.ps,
                 stringsAsFactors = FALSE)

colnames(surv.ps) <- c('Species', 'Census', 'K', 'p1',
                       'r1', 'p2', 'r2', 'thresh')
rownames(surv.ps) <- NULL

library(xtable)
sv.tab <- print(xtable(surv.ps), include.rownames = FALSE,
                digits = 3)

# plot the chains - make sure they are well mixed
par(mfrow = c(3,2))
```

```
lapply(s.params[[2]], function(x){
  apply(x[ ,2:5], 2, plot, type = 'l')
})
```

IPMs

```
################################################################################
### Script to make a vec-permutation matrix
################################################################################
rm(list = ls())
########################
### LIBRARIES ###
########################
library(Matrix)
library(MASS)

#######################
### FUNCTIONS ###
#######################
source(file = 'Source/IPM_construction_analysis.R')

#########################
### DATA ###
#########################
site <- 'bci'
sp.name <- 'calolo'
data.ls <- readRDS(file = sprintf('Output/%s_%s.RData',
                                  site, sp.name))

data.mat <- data.ls$data.mat
sp.latin <- data.ls$sp.latin[ ,1]
years <- data.ls$years

n.census <- length(grep('dbh', colnames(data.mat)))
n.intervals <- n.census - 1

q <- 0.95  # quantile for slow and fast growers

# IPM variables
sizes <- data.mat[ ,grep('dbh', colnames(data.mat))]
L <- min(sizes, na.rm = TRUE)*0.9  # smallest size
U <- max(sizes, na.rm = TRUE)*1.2  # largest size
S <- 1000  # number of size bins
h <- (U-L)/S  # size bin width
G <- 2  # number of growth distributions
meshpts <-  L + (1:S)*h -h/2  # midpoints of size bins
boundary <-  L + c(0:S)*h  # boundary points of size bins

# load the mcmc outputs for growth and survival parameters
load(file = sprintf('Output/%s_growthps_%s.RData',
                    site, sp.name))
load(file = sprintf('Output/%s_survps_%s.RData',
```

```
                    site, sp.name))

# increment threshold- this is where the two
# growth distributions meet
incr.thresholds <- g.params[[3]]

# make Mblock - the matrices describing size dependent
# transitions between growth distributions
# in this example transitions between distribtuions are 0
# so we can make a single GxG matrix and use the kronecker product
# with the Identity matrix of S.
# if transition probs are size dependent make a GxG matrix for each size
# (1...S) and then arrange as a block diagonal.
tt <- matrix(c(1,0,0,1), ncol = 2, byrow = TRUE)
Mblock <- kronecker(diag(rep(1, S)), tt)

# loop through census intervals and make an IPM for each.
# just using median parameter values here
# for demonstration - no parameter uncertainty

for(census in 1:n.intervals){

  # format parameters
  small.surv <- s.params[[1]][census, c('K', 'p1', 'r1')]
  big.surv <- s.params[[1]][census, c('K', 'p2', 'r2')]
  s.thresh <- s.params[[1]][census, 'thresh']
  alphas <- g.params[[1]][census, grep('alpha',
                                       colnames(g.params[[1]]))]
  betas <- g.params[[1]][census, grep('beta',
                                      colnames(g.params[[1]]))]
  incr.threshold <- incr.thresholds[census]

  # list of parameters
  m.par <- list(
    # survival
    surv.small  =  small.surv,
    surv.big    =   big.surv,
    surv.thresh = s.thresh,
    # growth
    g.slow = c(alphas[1], betas[1]),
    g.fast = c(alphas[2], betas[2]),
    # movement between growth distributions
    Mblock = Mblock,
    # class width
    h = h,
    # boundary
    boundary = boundary,
    # quantile for the two growth distributions
    q = 0.95,
    # increment threshold
    incr.thresh = incr.threshold
  )
```

```
  z1 <- z <- meshpts
  # make the kernel describing transitions between sizes and
  # growth distributions
  P.kernel <-  p_z1z(z1, z, m.par, G = 2, S = 1000)

  ### GET DIAGNOSTICS ###
  thresh <- 100  # get passage times to this size
  # and occupancy times below and above this size

  # occupancy
  occupancy.small <- get.occupancy.times(size.range =
                                          c(L, thresh),
                                        P.kernel, S, G, meshpts,
                                        G.int = seq(G))

  occupancy.big <- get.occupancy.times(size.range =
                                        c(thresh + 1, U),
                                      P.kernel, S, G,
                                      meshpts,
                                      G.int = seq(G))

  # passage times
  passage.times  <- get.passage.time(thresh, meshpts,
                                    c(1,2), P.kernel, S, G)

  # longevities
  longevities <- get.longevity(S, G, P.kernel)

  save(occupancy.big, occupancy.small, passage.times,
       longevities, P.kernel,
       file = sprintf('Output/IPM_%s_%s_%d.RData',
                      site, sp.name, census))

  print(census)
}
```

Plots

```
################################################################
### PLOTTING ###
################################################################
rm(list = ls())
############################
### LIBRARIRES ###
#############################
library(RColorBrewer)
library(doParallel)
library(foreach)

##########################
### FUNCTIONS ###
###########################
```

```
source("Source/IPM_construction_analysis.R")

###########################
### DATA ###
###########################
site <- 'bci'
sp.name <- 'calolo'
data.ls <- readRDS(file = sprintf('Output/%s_%s.RData',
                                  site, sp.name))

data.mat <- data.ls$data.mat
sp.latin <- data.ls$sp.latin[ ,1]
years <- data.ls$years
n.census <- length(grep('dbh', colnames(data.mat)))
n.intervals <- n.census - 1

q <- 0.95  # quantile for slow and fast growers

sizes <- data.mat[ ,grep('dbh', colnames(data.mat))]
L <- min(sizes, na.rm = TRUE)*0.9
U <- max(sizes, na.rm = TRUE)*1.2
S <- 1000
h <- (U-L)/S
G <- 2
meshpts <-  L + (1:S)*h -h/2 # midpoints
boundary <-  L + c(0:S)*h  # boundary points of mesh cells

# load the mcmc outputs for growth and survival parameters
load(file = sprintf('Output/%s_growthps_%s.RData',
                    site, sp.name))
load(file = sprintf('Output/%s_survps_%s.RData',
                    site, sp.name))

#####################################
### SURVIVAL ###
####################################
cols <- brewer.pal(9, 'YlGn')[4:(n.intervals+4)]

#pdf(file = sprintf('Figures/Vitals_%s.pdf', sp.name),
#   width = 10, bg = 'white')

par(mfrow = c(2,1), oma = c(0,0,2,0),
    mar = c(4,5,2,1))

# plots of SURVIVAL in each census
thresh <- s.params[[1]][1, 7]
small.surv <- s.params[[1]][1, 1:3]
big.surv <- s.params[[1]][1, 4:6]

# for plotting make U bigger- to show senescence
U <- U*1.25
size.1a <- seq(thresh) # to the threshold
size.2a <- seq(max(size.1a)+1, U) # ensures no overlap
```

```
s.survs <- s_z(size.1a, params = small.surv)
b.survs <- s_z(size.2a, params = big.surv)

plot(seq(U), c(s.survs, b.survs),
     main = '',
     font.main =1, las = 1, bty = 'l',
     lwd = 2, col = cols[1], ylim = c(0, 1), type = "l",
     xlab = '', ylab = '',
     cex.main = 1.3, cex.axis = 1.3)

legend('bottomleft', col = cols[1:n.intervals], lwd = 2,
       legend = years[1:(length(years)-1)], bg = 'white', bty = 'n')

# lines to show the threshold in each census interval
thresh.tmp <- s.params[[1]][ ,7]
abline(v = thresh.tmp, lty = 3)

# plot the uncertainty as grey polygons
for(census in 1:(n.intervals)){
  surv.chains.t <- s.params[[2]][[census]]

  uncert.mat <- matrix(NA, nrow(surv.chains.t),
                       U)
  for(tt in 1:dim(surv.chains.t)[1]){
    s.survs <- s_z(size.1a, params = surv.chains.t[tt,1:3])
    b.survs <- s_z(size.2a, params = surv.chains.t[tt,c(1, 4,5)])
    uncert.mat[tt, ] <- c(s.survs, b.survs)
  }

  us <- apply(uncert.mat, 2, quantile,
              c(0.025, 0.25, 0.5, 0.75, 0.975))

  xs <- seq(U)
  polygon(x = c(xs, rev(xs)), y = c(us[2, ], rev(us[4, ])),
          col = 'lightgrey', border = 'lightgrey')
}

# now add the lines of median survival in each census interval
for(census in 1:(n.intervals)){
  small.surv <- s.params[[1]][census, 1:3]
  big.surv <- s.params[[1]][census, 4:6]

  s.survs <- s_z(size.1a, params = small.surv)
  b.survs <- s_z(size.2a, params = big.surv)

  points(seq(U), c(s.survs, b.survs),
         lwd = 3, col = cols[census], type = "l")
}

mtext('DBH (mm)', side = 1, line = 2.5, cex = 1.4)
mtext('Survival probability', side = 2, line = 3,
      cex = 1.4)
mtext(bquote(''~italic(.(sp.latin))),
```

```
      side = 3, line = 1, cex = 1.5)

########################
### GROWTH ###
########################
# override other function - want a simpler version for plots
mix.gamma <- function(x, gam.pars) {
  slow.prob <- dgamma(x, gam.pars[1], gam.pars[3])
  fast.prob <- dgamma(x, gam.pars[2], gam.pars[4])
  return(tot.prob <- 0.95 * slow.prob + 0.05 * fast.prob)
}

cols <- brewer.pal(9, 'YlGn')[4:(n.intervals+4)]
cols.u <- brewer.pal(9, 'YlOrRd')[4:(n.intervals+3)]
cols.l <- brewer.pal(9, 'YlGnBu')[4:(n.intervals+3)]

cols.u.light <- make.ramp(cols.u, transp = 40,
                          levels = length(cols.u))
cols.l.light <- make.ramp(cols.l, transp = 40,
                          levels = length(cols.l))

load(sprintf('Output/%s_growthps_%s.RData',
             site, sp.name))

colgs <- ifelse(is.na(g.params[[1]][1,1]), 'white', cols.l[1])
colgf <- ifelse(is.na(g.params[[1]][1,1]), 'white', cols.u[1])

# range of increments to plot
xx <- seq(0.01, 25, 0.05)

slow.uncert <- fast.uncert <- vector('list', n.intervals)

# first get uncertainty
for(census in 1:(n.intervals)){
  gr.chains.t <- g.params[[2]][[census]]

  uncert.mat.sl <- uncert.mat.fs <- matrix(NA, nrow(gr.chains.t),
                                           length(xx))

  for(tt in 1:dim(gr.chains.t)[1]){

    slw <- dgamma(xx, gr.chains.t[tt,1], gr.chains.t[tt,3])
    fst <- dgamma(xx, gr.chains.t[tt, 2], gr.chains.t[tt, 4])
    uncert.mat.sl[tt, ] <- slw
    uncert.mat.fs[tt, ] <- fst
  }

  slow.uncert[[census]] <- apply(uncert.mat.sl, 2, quantile,
                                 prob = c(0.25, 0.5,  0.75))
  fast.uncert[[census]] <- apply(uncert.mat.fs, 2, quantile,
                                 prob = c(0.25, 0.5, 0.75))
}
```

```
ymax <- max(cbind(do.call(rbind, slow.uncert),
                  do.call(rbind, fast.uncert)), na.rm = TRUE)
tmp <- strsplit(sp.latin, ' ')

plot(xx, slow.uncert[[1]][2, ], type = 'l', col = colgs,
     lwd = 2, ylim = c(0, ymax),
     las = 1, cex.axis = 1.3,
     xlab = '', ylab = '', bty = 'l',
     main = '', yaxt = 'n')

# uncertainty
for(census in 1:(n.intervals)){
  polygon(x = c(xx, rev(xx)), y = c(slow.uncert[[census]][1, ],
                                    rev(slow.uncert[[census]][2, ])),
          col = 'lightgrey', border = 'lightgrey')
  polygon(x = c(xx, rev(xx)), y = c(fast.uncert[[census]][1, ],
                                    rev(fast.uncert[[census]][2, ])),
          col = 'lightgrey', border = 'lightgrey')
}

# and the median of uncertainty
mapply(function(x, y) points(xx, x[2, ], type = 'l', col = y, lwd = 2),
       x = slow.uncert,
       y = cols.l)
mapply(function(x,y) points(xx, x[2, ], type = 'l', col = y, lwd = 2),
       x = fast.uncert,
       y = cols.u)

mtext('DBH increment (mm)', side = 1, line = 2.5,
      cex = 1.4)
mtext('Density', side = 2, line = 3, cex = 1.4)

# now an insert of the mixed distribution
# use median params from all censuses combined
all.uncert <- do.call(rbind, g.params[[2]])
gam.pars <- apply(all.uncert, 2, median)
# mixed gamma distribution weighted by proportion of
# the population in each distribution
y <- mix.gamma(xx, gam.pars)
xlim <- c(min(xx), max(xx))
# Create and plot a spline function that interpolates between the points.
fn <- splinefun(xx, y)

# get the threshold which is 0.95 quantile of all growth
incr.threshold <- g.params[[3]][1]  # use first increment threshold as example
seq.lo <- which(xx < incr.threshold)
seq.hi <- which(xx >= incr.threshold)

u <- par("usr")
v <- c(
  grconvertX(u[1:2], "user", "ndc"),
  grconvertY(u[3:4], "user", "ndc")
)
```

```
v <- c( (v[1]+v[2])/2, v[2], (v[3]+v[4])/2, v[4] )

par(fig = v, new = TRUE, mar = c(0,0,0,0))
curve(fn, xlim[1], incr.threshold, n = 1000, main = '',
      pch = 18, xlim = range(xx),
      xlab = '', bty = 'l', ylim = c(0,0.5),
      cex.axis = 0.01)
points(xx[seq.lo], y[seq.lo], pch = 18,
       col = cols.l[n.intervals-2], cex = 0.3)
points(xx[seq.hi], y[seq.hi], pch = 18,
       col = cols.u[n.intervals-2], cex = 0.3)
abline(v = incr.threshold, lty = 2, col = 'darkgrey')


#dev.off()

######################################################################
### IPM OUTPUTS ###
# just plotting most recent census interval here
######################################################################

# reset U
U <- max(sizes, na.rm = TRUE)*1.2

#pdf(file = 'Figures/IPM_outputs.pdf', bg = 'white')
cols <- brewer.pal(5, 'Dark2')

par(mar = c(5,5,4,1),
    oma = c(0,0,0,0), mfrow = c(2,2))

census <- n.census-1
load(file = sprintf('Output/IPM_%s_%s_%d.RData',
                    site, sp.name, census))

G2pts <- matrix(passage.times$mu, ncol = S*2)
y.max <- max(G2pts)*1.2
g.thresh <- 100
mesh.g.thresh <- (which(abs(meshpts - g.thresh) ==
                          min(abs(meshpts-g.thresh)))) + 2

plot(meshpts[1:mesh.g.thresh], G2pts[1:mesh.g.thresh],
     type = 'l', xlab = '',
     ylab = '', cex.axis = 1.2, cex.main = 1.2, las = 1,
     main = sprintf('Passage Times to %d mm', round(g.thresh)),
     col = cols[1], cex.lab = 1.4,
     ylim = c(0, y.max), lwd = 2.5, font.main = 1,
     bty = 'l')

# add passage times in the fast growth distribution
points(meshpts[1:mesh.g.thresh],
       G2pts[(S+1): (S+mesh.g.thresh)], type = 'l',
       col = cols[2], lwd = 2.5)
legend('topright', legend = seq(G), col = cols[1:G], lwd = 2.5,
```

```
       title = 'Growth Distribution',
       lty = c(1,1), cex = 1.2, bty = 'n')

# LE
G2le <- matrix(longevities$mu, ncol = S*2)
y.max <- max(G2le)*1.25

plot(meshpts, G2le[1:S], type = 'l', xlab = '',
     ylab = '', cex.lab = 1.4,
     main = 'Life expectancy', col = cols[1],
     ylim = c(0, y.max), lwd = 2.5, font.main = 1, bty = 'l',
     las = 1, cex.axis = 1.2, cex.main = 1.2)
# life expectancies of fast growing trees
points(meshpts, G2le[(S+1): (S*2)],
       type = 'l', col = cols[2], lwd= 2.5)

# Occupancy times
o.small.sizes <- matrix(occupancy.small$o.sizes, ncol = S*2)
o.big.sizes <- matrix(occupancy.big$o.sizes, ncol = S*2)

plot(meshpts[1:mesh.g.thresh],
     o.small.sizes[1, 1:mesh.g.thresh],
     type = 'l', col = cols[1],
     xlab = '', bty = 'l', las = 1, cex.axis = 1.2,
     ylab = '', xlim = c(0, g.thresh*1.1),
     main = sprintf('Occupancy < %d mm', round(g.thresh)),
     lwd = 2, ylim = c(0, max(o.small.sizes[1,])),
     font.main = 1, cex.main = 1.2)
points(meshpts[1:mesh.g.thresh],
       o.small.sizes[1, (S+1):(S+mesh.g.thresh)],
       type = 'l', col = cols[2], lwd = 2)
points(meshpts[1:mesh.g.thresh],
       o.small.sizes[2, 1:mesh.g.thresh], type = 'l',
       col = cols[3], lwd = 2)
points(meshpts[1:mesh.g.thresh],
       o.small.sizes[2, (S+1):(S+mesh.g.thresh)], type = 'l',
       col = cols[4], lwd = 2)
legend('topright', lwd = 2, col = cols, cex = 1.2,
       legend = c('Slow | start slow', 'Slow | start fast',
                  'Fast | start slow', 'Fast | start fast'),
       bty = 'n')

plot(meshpts, o.big.sizes[2, 1:S],
     type = 'l', col = cols[4],
     xlab = '', cex.axis = 1.2,
     ylab = '', bty = 'l', las = 1,
     main = sprintf('Occupancy >= %d mm', round(g.thresh)),
     lwd = 2, font.main = 1, cex.main = 1.2,
     ylim = c(0, max(o.big.sizes)*1.1))
points(meshpts, o.big.sizes[2, (S+1):(S*2)],
       type = 'l', col = cols[5], lwd = 2)
legend('topright', lwd = 2, col = cols[c(3,4)], cex = 1.2,
       legend = c('Fast | start slow', 'Fast | start fast'),
```

```
       bty = 'n')

mtext('DBH (mm)', side = 1, line = 1.5,
      outer = TRUE, cex = 1.3)
mtext('Time (years)', side = 2, line = 1.5,
      outer = TRUE, cex = 1.3)


#dev.off()
```

Functions

Data preparation functions

```
# correct dbh measurements for all censuses at once
correct.dbh <- function(dbhs,
                        sd2 = 25.6, times = 5,
                        sda = 0.927, sdb = 0.0038,
                        give.up = 20000){

  dim.col <- dim(dbhs)[2]
  # propose new parameters
  # size dependent growth correction
  suppressWarnings(dbh.stars <-  matrix(rnorm(length(dbhs), dbhs,
                                          (sda + sdb * dbhs)),
                                     ncol = dim.col, byrow = FALSE))

  # get the increments
  diffs <- t(apply(dbh.stars, 1, diff))
  if(dim.col == 2){
    diffs <- t(diffs)
  }

  # need to correct everything negative AND positive since
  # just correcting negative introduces a bias
  # so to start everything gets jittered according to the error distribution
  # things that are still negative get repeatedly jittered until positive
  neg <- seq(nrow(dbh.stars))

  ct <- 0
  # while anything is still negative keep going
  # until give.up to try and correct them
  while(length(neg)>1 & ct < give.up) {

    # propose new sizes for negative stems/increments
    suppressWarnings(dbh.stars[neg, ] <-
                       matrix(rnorm(length(dbhs[neg ,]), dbhs[neg ,],
                                    (sda + sdb * dbhs[neg ,])),
                              ncol = dim.col, byrow = FALSE))
    # new increments for the new sizes
    diffs[neg, ] <- t(apply(dbh.stars[neg, ], 1, diff))
    # which ones are still negative
```

```
    neg.incrs <- which(apply(diffs, 1, function(x) any(x <= 0)) == TRUE)
    neg.stems <- which(apply(dbh.stars, 1, function(x) any(x <= 0)) == TRUE)
    neg <- unique(c(neg.incrs, neg.stems))
    ct <- ct + 1

    if(ct %% 1000 == 0){
      print(sprintf('%d: Negative stems/increments: %d', ct, length(neg)))
      print(length(which(is.na(dbh.stars))))
    }
  }
  # at give up... if there are still negative stems/increments
  if(length(neg) > 0) {

    # first correct negative sizes
    dbh.stars[which(dbh.stars < 0)] <- rnorm(length(which(dbh.stars < 0)), 10, 0.5)

    # now get new diffs
    diffs <- t(apply(dbh.stars, 1, diff))
    if(dim.col == 2){
      diffs <- t(diffs)
    }

    # which rows still have negative growths
    neg <- which(apply(diffs, 1, function(x) any(x <= 0)) == TRUE)
    # if a stem has a negative incrment AND one or more positive increment
    # then make the negative the mean of the positives
    # if it has only negative increments then sample from
    # ALL stems.
    if(length(neg) > 0){
      tmpll <- matrix(diffs[neg, ], ncol = ncol(diffs))

      diffs[neg, ] <- t(apply(tmpll, 1,
                              function(x) replace(x, which(x < 0),
                                                  ifelse(length(which(x > 0)) > 0,
                                                         mean(x[x > 0], na.rm = TRUE),
                                                         sample(diffs[diffs > 0 & !is.na(diffs)],
                                                                length(which(x<=0)),
                                                                replace = TRUE)))))
      # adjust the sizes to take account of the new increments
      # NB some don't have a size in the first few years
      dbh.stars <- t(mapply(make.new.sizes, sizes = split(dbh.stars, row(dbh.stars)),
                            incrs = split(diffs, row(diffs))))

    }
  }
  n.sampled <- length(neg)  # how many were not corrected before give.up
  return(list(dbh.stars = dbh.stars, n.sampled = n.sampled))
}

# get new dbh measurements based on estimates of error rates
propose.incr <- function(dbhs, sda = 0.927, sdb = 0.0038) {
  dbhs.star <- rnorm(length(dbhs), dbhs, (sda + sdb*dbhs))
  return(dbhs.star)
```

```
}


# find obvious outliers - those that grew more than 75 mm
# or shrank more than 25% of original DBH
# from the other measurements
find.errors <- function(stem.vec){
  stem <- stem.vec[grep('dbh', names(stem.vec))]
  diffs <- stem.vec[grep('incr', names(stem.vec))]
  time.vec <- stem.vec[grep('time', names(stem.vec))]
  diffs <- diffs/time.vec  # make the increments annual

  #  returns true if the stem shrank more than 25% of original size
  # or grew more than 75 mm in a single year
  out <- ifelse(any(diffs > 75, na.rm = TRUE), TRUE, FALSE)

  tmp.ll <- cbind(stem[1:(length(stem)-1)], stem[2:length(stem)])
  out <- ifelse(any(tmp.ll[,2] < 0.75*tmp.ll[,1], na.rm = TRUE), TRUE, out)

  # can't correct if there are only 2 measurements
  if(length(which(!is.na(diffs))) < 2){
    out <- FALSE
  }
  return(out)
}


# correct those obvious outliers - make them the mean of measurements either side
# or if they are first or last make them second/penultimate -/+ growth from closest
# census - only works when there are >2 measurements
correct.errors <- function(stem.vec){
  full <- stem.vec
  start <- as.numeric(stem.vec[grep('dbh', names(stem.vec))])
  stem <- start[!(is.na(start))]  # get rid of NAs
  if(length(stem) > 2){ # can only correct if there are more than two measurements

    # get the difference between each measurement and every other
    # measurement
    co <- cbind(stem, rep(1, length(stem)))
    dists <- dist1(co)
    outlier <- which(rowMeans(dists) == max(rowMeans(dists)))[1]

    if(outlier == 1){
      stem[outlier] <- stem[2] - diff(stem)[2]
    }else{
      if(outlier == length(stem)){
        stem[outlier] <- stem[length(stem) -1] + diff(stem)[length(stem) - 2]
      }else{
        stem[outlier] <- mean(c(stem[outlier- 1], stem[outlier + 1]))
      }
    }
  }
```

```
  start[!is.na(start)] <- stem
  full[grep('dbh', names(full))] <- start
  return(full)
}


# remove trees that are more than twice as big as the
# 99th quantile of size
remove.too.big <- function(sp.df){
  sizes <- sp.df[ ,grep('dbh', colnames(sp.df))]
  q.99 <- quantile(sizes, prob = 0.99, na.rm = TRUE)
  too.big <- which(apply(sizes, 1, function(x) ifelse(any(x > q.99*2, na.rm = TRUE),
                                                       TRUE, FALSE)) == TRUE)
  if(length(too.big) > 0){
    sp.df <- sp.df[-too.big, ]
  }
  return(sp.df)
}


make.new.sizes <- function(sizes, incrs){
  first <- which(!is.na(sizes))[1]
  if(first != length(sizes)){
    out <- cumsum(c(sizes[first], incrs[first:length(incrs)]))
    out <- c(rep(NA, first-1), out)
  }else{
    out <- sizes
  }
  return(out)
}
```

IPM Construction and Analysis

```
######################################################
### Functions for IPM construction and analysis ###
#####################################################
# make nice colour palettes
make.ramp <- function(tmpcol, levels = 10, transp = NA) {
  col.out <- colorRampPalette(tmpcol)(levels)
  if(!is.na(transp)) {
    col.out <- paste(col.out, transp, sep = "")
  }
  return(col.out)
}

# make transparent colours
make.transp <- function(tmpcol, transp = 50) {
  col.out <- paste(tmpcol, transp, sep = "")
  return(col.out)
}
```

```
# Makes the vec-permutation matrix
make.vec.mat <- function(G = 2, S = 1000) {
  n.t <- matrix(seq(G * S), ncol = 1) # ex. pop vector
  n.tk <- matrix(n.t, ncol = G, byrow = FALSE)
  n.tk.new <- matrix(t(n.tk), ncol = 1, byrow = FALSE)

  comp <- cbind(n.t, n.tk.new)
  vec.mat.tmp <- matrix(0, G*S, G*S)   # empty transition matrix
  vec.mat <- replace(vec.mat.tmp, comp, 1)
  return(vec.mat)
}

# Given growth coordinates get the mixed gamma distribution

# 1, combined and weighted Gamma distributions
mix.gamma <- function(x, m.par){
  prop <- c(m.par$q, (1 - m.par$q))
  slow <- m.par$g.slow
  fast <- m.par$g.fast
  slow.prob <- dgamma(x, slow[1], slow[2]) * prop[1]
  fast.prob <- dgamma(x, fast[1], fast[2]) * prop[2]
  return(tot.prob <- slow.prob + fast.prob)
}

cdf <- function(fn, min_x, max_x){
  # Returns a cumulative distribution function for a non-negative function over a given range.
  f <- function(x){
    y <- rep(NA, length(x))
    total_area_under_curve <- integrate(fn, min_x, max_x)$value
    apply_fn <- function(xval){integrate(fn, min_x, min(xval, max_x))$value}
    y <- sapply(x,
                FUN=apply_fn) / total_area_under_curve
    y <- cummax(y)
    y[x < min_x] <- 0
    y[x > max_x] <- 1
    return(y)
  }
  return(f)
}


# make cdf for each distribution then sample from it.
# if x is outside of range set to 0
get.cdfs <- function(meshpts, m.par){

  S <- length(meshpts)
  boundary <- m.par$boundary
  h <- m.par$h
  incrs.pos <- boundary[2:length(boundary)] - meshpts[1] # DBH change from one size class to the next
  # no. size classes to jump that gives threshold between slow and fast incrs
  thresh <- which.min(abs(incrs.pos - m.par$incr.thresh))

  x1 <- incrs.pos[1:(thresh-1)]
```

```
  x2 <- incrs.pos[thresh: length(incrs.pos)]
  y1 <- mix.gamma(x1, m.par)
  y2 <- mix.gamma(x2, m.par)
  xlim <- c(min(x1), max(x2))

  fn.1 <- splinefun(x1, y1)
  fn.2 <- splinefun(x2, y2)
  cdf_fn_slow <- cdf(fn.1, x1[1], x1[length(x1)])
  cdf_fn_fast <- cdf(fn.2, x2[1], x2[length(x2)])

  mix.cdf <- vector('list', 2)
  mix.cdf[[1]] <- vector('list', 2)
  mix.cdf[[1]][[1]] <- incrs.pos
  mix.cdf[[1]][[2]] <- cdf_fn_slow(x1)
  # for the matrix size sensitivity large meshpts can result in NaN so fix this
  mix.cdf[[1]][[2]] <- ifelse(is.nan(mix.cdf[[1]][[2]]),
                              1, mix.cdf[[1]][[2]])
  mix.cdf[[2]][[1]] <- incrs.pos
  mix.cdf[[2]][[2]] <- cdf_fn_fast(x2)
  names(mix.cdf[[1]]) <- c('x', 'y')
  names(mix.cdf[[2]]) <- c('x', 'y')
  names(mix.cdf) <- c('slow', 'fast')

  # a cdf has to be monotonically increasing and bounded between 0 and 1.
  mix.cdf$fast$y[which(mix.cdf$fast$y > 1)] <- 1
  mix.cdf$slow$y[which(mix.cdf$slow$y > 1)] <- 1

  mix.cdf$slow$y <- c(mix.cdf$slow$y,
                      rep(1, (S - length(mix.cdf$slow$y))))
  fs.gr <- rep(0, S)
  fs.gr[thresh:(thresh-1 + length(mix.cdf$fast$y))] <- mix.cdf$fast$y
  mix.cdf$fast$y <- fs.gr

  return(mix.cdf)
}

pg_z1z.ll <- function(z, z1, mix.cdf){
  incr <- z1 - z
  u <- mix.cdf$y[which.min(abs(mix.cdf$x - incr))]
  return(u)
}


# cumulative probability of moving from one size to another
pg_z1z <- function(z, z1, params.g){
  u <- pgamma((z1 - z), params.g[1], params.g[2])
  return(u)
}

# make the G matrix for each growth distribution
g_z1z <- function(meshpts, m.par, G = 2, S = 1000){

  boundary <- m.par$boundary
```

```
  h <- m.par$h
  incrs.pos <- boundary[2:length(boundary)] - meshpts[1] # DBH change from one size class to the next
  # no. size classes to jump that gives threshold between slow and fast incrs
  thresh <- which.min(abs(incrs.pos - m.par$incr.thresh))

  # get the mixed gamma distribution
  mix.cdf <- get.cdfs(meshpts, m.par)

  # cumulative slow
  vecs <- vector('list', length(meshpts))
  for(bb in 1:length(meshpts)){
    vecs[[bb]] <- sapply(boundary[2:length(boundary)],
                         function(x) pg_z1z.ll(meshpts[bb], x, mix.cdf$slow))
  }
  Gmat.sl <- do.call(cbind, vecs)

  # cumulative fast
  vecs <- vector('list', length(meshpts))
  for(bb in 1:length(meshpts)){
    vecs[[bb]] <- sapply(boundary[2:length(boundary)],
                         function(x) pg_z1z.ll(meshpts[bb], x, mix.cdf$fast))
  }
  Gmat.fs <- do.call(cbind, vecs)

  Gmats <- list(Gmat.sl, Gmat.fs)

  # actual prob of moving from one size to next
  Gmats <- lapply(Gmats, function(mat){
    apply(mat, 2, function(x) x[2:S] - x[1:(S-1)])})

  Gmats <- lapply(Gmats, function(mat){
    rbind(0, mat)})

  # deal with all 0s in slow mat (this happens when thresh is 1)
  if(all(colSums(Gmats[[1]]) == 0)){
    Gmats[[1]] <- diag(S)
    Gmats[[1]] <- rbind(0, Gmats[[1]][1:(S-1), ])
  }

  # any that have colsum of 0 need 1 as last entry
  Gmats <- lapply(Gmats, function(mat){
    cs <- which(colSums(mat) == 0);
    mat[S, cs] <- 1;
    mat
  })

  # now normalise remaining values
  Gmats <- lapply(Gmats, function(mat){
    mat <- apply(mat, 2, function(x) x/sum(x));
    mat})

  return(Gmats)
}
```

```
# predict survival for size z
s_z <- function(z, params.s, time = 1) {
  K <- params.s[1]
  p <- params.s[2]
  r <- params.s[3]
  # Linear predictor parameters
  pred.y <-  (K /
               (1 + exp(-(r * ((z - p) )))))  ^ (time)
  return(pred.y)
}

# make a survival vector
s_z_vec <- function(z, m.par, time = 1){

  surv.thresh <- m.par$surv.thresh
  small.params <- m.par$surv.small
  big.params <- m.par$surv.big

  small <- z[which(z < surv.thresh)]
  big <- z[which(z >= surv.thresh)]

  p.small <- s_z(small, small.params)
  p.big <- s_z(big, big.params)

  surv <- c(p.small, p.big)

  return(surv)
}

# make the vec permutation P matrix
p_z1z <- function(z1, z, m.par, G = 2, S = 1000){
  Mblock <- m.par$Mblock

  # GROWTH -
  Gmats <- g_z1z(z1, m.par, G, S)

  # Survival
  Svec <- s_z_vec(z1, m.par)

  Pmats <- vector('list', G)

  Pmats <- lapply(Gmats, function(x) t(t(x)*Svec))
  Pmats <- lapply(Pmats, function(x) {x[S,S] <- 0;
  return(x)})

  vec.mat <- make.vec.mat(G, S)  # make the vec permutation matrix
  vec.mat <- Matrix(vec.mat, sparse = TRUE)

  Pblock <- bdiag(Pmats) # Within growth environment transitions on the diagonal.

  Pkernel <- t(vec.mat) %*% Mblock %*% vec.mat %*% Pblock

  return(Pkernel)
```

```
}



#################################################################################
# DIAGNOSTICS ###

# 1. OCCUPANCY TIMES
get.occupancy.times <- function(size.range = c(meshpts[1], meshpts[2]), Ptilde, S, G, meshpts,
                               G.int = seq(G)){

  I <- diag(S*G)  # Identity matrix
  Ptilde <- as.matrix(Ptilde)
  Ntilde <- ginv(I - Ptilde)  # Fundamental matrix of Utilde -
  # Ntilde[i,j] is expected occupancy time in state i | starting in state j

  # size bins corresponding to size ranges of interest
  min.c <- which(abs(meshpts- size.range[1]) == min(abs(meshpts- size.range[1])))
  max.c <- which(abs(meshpts - size.range[length(size.range)]) ==
                   min(abs(meshpts - size.range[length(size.range)])))

  cs <- rep(0, S)  # S x 1 vector indicating which sizes classes of interest
  cs[seq(min.c, max.c, 1)] <- 1

  # o.sizes[i,j] is the sum of occupancy in each size class of interest for growth
  # group i, given starting state j (combination of sizes and growth)
  o.sizes <- kronecker(Diagonal(G), t(cs)) %*% Ntilde  # G x SG

  cg <- rep(0, G)
  cg[G.int] <- 1  # G x 1 vector indicating groups of interest

  # o.growth[i,j] is the sum of occupancy in each growth class of interest for size
  # class i, given starting state j (combination of sizes and growth)
  o.growth <- kronecker(t(cg), Diagonal(S)) %*% Ntilde

  # occupancy in each size/growth group of interest
  o.both <- kronecker(t(cg), t(cs)) %*% Ntilde

  return(list(Ntilde = Ntilde, o.sizes = o.sizes, o.growth = o.growth,
              o.both = o.both))
}


# get observed size distributions
get.observed.size.dist <- function(size.vec, S, boundary){
  complete.size <- size.vec[!is.na(size.vec)]
  tmp <- cut(complete.size, breaks = c(0,boundary, Inf), labels = FALSE,
             include.lowest = TRUE)
  S.y <- rep(0, S)  # observed size distribution
  S.y[as.numeric(names(table(tmp)))] <- as.numeric(table(tmp))
  return(S.y)
}
```

```
# 2. Mixed distribution
# i.e. occupancy times for a set of individuals mixed among sizes
# and/or groups


# gets the occupancy times of a cohort
# i.e. individuals start in different growth/size groups
get.occupancy.mixed <- function(size.vec, S, boundary, Ptilde, quant, G){

  n.vec <- get.observed.size.dist(size.vec, S, boundary)
  n.vec <- c(n.vec*quant, n.vec*(1 - quant))  # separate into growth classes
  n.vec <- n.vec/sum(n.vec)  # normalise
  I <- diag(S*G)  # Identity matrix
  Ptilde <- as.matrix(Ptilde)
  Ntilde <- ginv(I - Ptilde)  # Fundamental matrix of Utilde
  o.mix <- Ntilde %*% n.vec  # occupancy in each state
  return(o.mix)
}


# SURVIVORSHIP
get.survivorship <- function(x, S, G, Ptilde){

  Ptilde <- as.matrix(Ptilde)
  l.tilde <- t(t(rep(1, (S*G))) %*% as.matrix(Ptilde%^%x, (S*G), (S*G)))
  # ith entry is survivors to age x of a cohort in state i at time 0.
  return(l.tilde)

}

# survivorship of a cohort with mixed distribution of sizes and growth classes
get.mixed.survivorship <- function(x, size.vec, S, G, boundary, quant, Ptilde){

  Ptilde <- as.matrix(Ptilde)
  # survivorship of cohort from state j after x years
  l.tilde <- t(t(rep(1, (S*G))) %*% as.matrix(Ptilde%^%x, (S*G), (S*G)))

  n.vec <- get.observed.size.dist(size.vec, S, boundary)  # size distribution
  n.vec <- n.vec/sum(n.vec)
  # survivorship of mixed cohort after x years, by growth class
  mixed.surv <- t(kronecker(diag(G), n.vec)) %*% l.tilde[,1]

  return(mixed.surv)

}


# longevity statistics
get.longevity <- function(S, G, Ptilde){
  I <- diag(S*G)  # Identity matrix dimensions of Utilde
  Ptilde <- as.matrix(Ptilde)
  Ntilde <- ginv(I - Ptilde)  # Fundamental matrix of Utilde
  # moments
```

```
  m1 <- t(rep(1, S*G)) %*% Ntilde
  m2 <- m1 %*% (2*Ntilde - diag(S*G))
  m3 <- m1 %*% (6*(Ntilde%*%Ntilde) - 6*Ntilde + diag(S*G))
  # variance
  var <- colSums(2 * Ntilde %*% Ntilde - Ntilde) - colSums(Ntilde) * colSums(Ntilde)

  return(list(mu = m1, var = var, m3 = m3))
}

# probability distribution of longevity
get.longevity.prob.dist <- function(ns, S, G, Ptilde){
  Ptilde <- as.matrix(Ptilde)
  p.eta <- sapply(ns, function(x)
    t(colSums(diag(S*G) - Ptilde) %*% (Ptilde %^% (x-1))))
  return(p.eta)
}



# passage times to sets of states
get.passage.time <- function(size.range, meshpts, G.int, Ptilde, S, G){

  # Define the set of states - move from actual sizes to corresponding size bins
  min.c <- which(abs(meshpts- size.range[1]) == min(abs(meshpts- size.range[1])))
  max.c <- which(abs(meshpts - size.range[length(size.range)]) ==
                  min(abs(meshpts - size.range[length(size.range)])))

  # vector of states of interest
  R <- c()
  for(g in 1:length(G.int)){
    R <- c(R, seq(min.c, max.c, 1) + S*(g-1))
  }

  Mtilde <- 1 - colSums(Ptilde)  # probability of death
  Mtilde[R] <- 0  # already in the absorbing state

  Pprime <- Ptilde
  Pprime[ ,R] <- 0  # those in the states of interest are in the absorbing state
  Pprime[R, ] <- 0

  Mprime <- Mtilde
  Mprime <- rbind(Mprime, colSums(Ptilde[R, ]))
  Mprime[2, R] <- 1

  Pprime <- as.matrix(Pprime)
  Ntilde.prime <- ginv(diag(G*S) - Pprime)
  Bprime <- Mprime %*% Ntilde.prime

  # CONDITIONAL MARKOV CHAIN
  Ptilde.c <- diag(Bprime[2,]) %*% Pprime %*% ginv(diag(Bprime[2,]))

  eta1 <- ginv(diag(G*S) - Ptilde.c)
```

```
  var <- colSums(2 * (eta1 %*% eta1) - eta1) - colSums(eta1) * colSums(eta1)

  m1 <- colSums(eta1)
  m1[R] <- 0

  return(list(mu = m1, var = var))
}
```

Stan Files

The following need to be saved as .stan files.

Growth

```
data {
  int<lower=0> N;       // no. of trees
  real incr[N];         // diameter increment in mm
  real groups[N];   // which growth groups
}

parameters {
  real<lower=0, upper=1000> alpha1;
  real<lower=0, upper=1000> alpha2;
  real<lower=0, upper=1000> beta1;
  real<lower=0, upper=1000> beta2;
}

model {
  for(i in 1:N)
  {
    if(groups[i] == 1)
      target += (gamma_lpdf(incr[i] | alpha1, beta1));
      else
        target += (gamma_lpdf(incr[i] | alpha2, beta2));
  }
}
```

Survival

```
data {
  int<lower=0> N;                       // no. of trees
  real<lower=0> dbh[N];                 // diameter in mm
  int<lower=0, upper=1> surv[N];      // survival of each tree, 1 for alive, 0 for dead
  real time[N];                         // time interval between two censuses
  real thresh;
  real lowr2;
  real upr2;

}
```

```
parameters {
  real<lower=0.01, upper=0.995> K;
  real<lower=-1*thresh, upper=thresh*0.75> p1;
  real<lower=0.0001, upper=0.25> r1;

  real<lower=max(dbh)*1.2, upper=2*(max(dbh))> p2;
  real<lower=lowr2, upper=upr2> r2;
}

model {
  real p;
  real theta;

  for(i in 1:N)
  {
    if(dbh[i] < thresh)
    {
      p = (K) / (1 + exp(-r1 * (dbh[i] - p1)));
      theta = p^time[i];
    }
    else
    {
      p =  (K) / (1 + exp(-r2 * (dbh[i] - p2)));
      theta = p^time[i];
    }
    surv[i] ~ bernoulli(theta);
  }
}
```
